# Evaluation of left intraventricular flow dynamics using the novel non-contrast HyperDoppler technique

**DOI:** 10.1093/ehjimp/qyag050

**Published:** 2026-03-19

**Authors:** Riccardo Beccari, Antonella Cecchetto, Vittorio Smarrazzo, Vittoria Miano, Mauro Pepi, Fabio Fazzari, Laura Stefani, Marco Corsi, Matteo Cameli, Marta Focardi, Concetta Zito, Giancarlo Trimarchi, Jaroslaw Kasprzak, Dominika Filipiak-Strzecka, Chiara Cogliati, Daniela Torzillo, Iolanda Aquila, Ettore Ventura, Jolanda Sabatino, Rosalba De Sarro, Marco Pepe, Pierluigi Incarnato, Giampaolo Bezante, Paolo Costa, Omar Prieto, Marco Maglione, Raphael Rascón-Sabido, Dario Gregori, Jagat Narula, Gianni Pedrizzetti, Donato Mele

**Affiliations:** Cardiac Unit, Department of Cardiac Thoracic Vascular Sciences and Public Health, University of Padova, Via Giustiniani 2, 35128 Padova, Italy; Cardiac Unit, Department of Cardiac Thoracic Vascular Sciences and Public Health, University of Padova, Via Giustiniani 2, 35128 Padova, Italy; Cardiac Unit, Buon Consiglio Fatebenefratelli Hospital, Napoli, Italy; Cardiac Unit, Buon Consiglio Fatebenefratelli Hospital, Napoli, Italy; Centro Cardiologico Monzino IRCCS, Milano, Italy; Centro Cardiologico Monzino IRCCS, Milano, Italy; Sports Medicine Center, University of Firenze, Firenze, Italy; Sports Medicine Center, University of Firenze, Firenze, Italy; Division of Cardiology, Department of Medical Biotechnologies, University of Siena, Siena, Italy; Division of Cardiology, Department of Medical Biotechnologies, University of Siena, Siena, Italy; Department of Clinical and Experimental Medicine, University of Messina, Messina, Italy; Department of Clinical and Experimental Medicine, University of Messina, Messina, Italy; Interdisciplinary Center for Health Sciences, Scuola Superiore Sant'Anna, Pisa, Italy; I Department of Cardiology, Medical University of Lodz, Lodz, Poland; I Department of Cardiology, Medical University of Lodz, Lodz, Poland; Internal Medicine, L.Sacco Hospital, ASST Fatebenefratelli-Sacco, Milano, Italy; Department of Biomedical and Clinical Sciences, University of Milano, Milano, Italy; Internal Medicine, L.Sacco Hospital, ASST Fatebenefratelli-Sacco, Milano, Italy; Cardiac Unit, University Hospital Renato Dulbecco, Catanzaro, Italy; Cardiac Unit, University Hospital Renato Dulbecco, Catanzaro, Italy; Pediatric Cardiology, Department of Experimental and Clinical Medicine, ‘Magna Graecia’ University of Catanzaro, Catanzaro, Italy; Pediatric Cardiology, Department of Experimental and Clinical Medicine, ‘Magna Graecia’ University of Catanzaro, Catanzaro, Italy; Cardiac Unit, San Michele Hospital, Maddaloni, Italy; Cardiac Unit, San Michele Hospital, Maddaloni, Italy; Cardiovascular Clinic, San Martino Hospital IRCCS, Genova, Italy; Cardiovascular Clinic, San Martino Hospital IRCCS, Genova, Italy; Faculty of Medicine, University of Buenos Aires, Argentina; GLM Esaote, Genova, Italy; Unidad de alta especialidad IMSS, Universidad Veracruzana, Veracruz, México; Unit of Biostatistics, Epidemiology and Public Health, Department of Cardiac, Thoracic and Vascular Sciences, University of Padova, Padova, Italy; Heart & Vascular Institute, University of Texas Health Science Center, Houston, TX, USA; Department of Engineering and Architecture, University of Trieste, Trieste, Italy; Cardiac Unit, Department of Cardiac Thoracic Vascular Sciences and Public Health, University of Padova, Via Giustiniani 2, 35128 Padova, Italy; Cardiac Unit, Ravenna33 Clinic, Ravenna, Italy

**Keywords:** HyperDoppler, intracardiac flow dynamics, vector flow mapping

## Abstract

**Aims:**

Recently, HyperDoppler, a colour Doppler-based ultrasound technique, has been introduced for left ventricular (LV) flow dynamics evaluation in clinical settings, but quantitative information derived from its use is limited. We aimed at establishing reference values and reproducibility of key HyperDoppler LV flow dynamics measures: vortex area, length, depth, intensity, and global kinetic energy dissipation (gKED) in normal subjects. We also explored the influence of physiological and echocardiographic variables on HyperDoppler measures.

**Methods and results:**

This multicentre, international study involved 13 echocardiographic laboratories and an independent echo corelab. A total of 467 normal subjects were enrolled and categorized by gender and age (20–39, 40–59, and 60–79 years): of these, 317 subjects were analysed at the corelab and 419 on-site using different colour Doppler cineloops. Corelab analysis yielded a median value (25th–75th percentile) of 25.0% (21.1–28.0%) for vortex area, 57.4% (53.3–62.6%) for vortex length, 34.3% (30.7–38.1%) for vortex depth, −31.9% (−28.0% to −35.8%) for vortex intensity, and 0.55 (0.44–0.70) for gKED. Minor age- and gender-related variations were noted in vortex length, depth, and gKED. Excellent reproducibility was shown for each HyperDoppler measure. Physiological behaviour of left intraventricular flow dynamics was adequately captured by the HyperDoppler quantitative measures.

**Conclusion:**

Reference values for left intraventricular flow dynamics were established using the HyperDoppler technique, which is reproducible and enables the assessment of intracardiac flow dynamics in clinical practice.

## Graphical Abstract

**Graphical Abstract:**
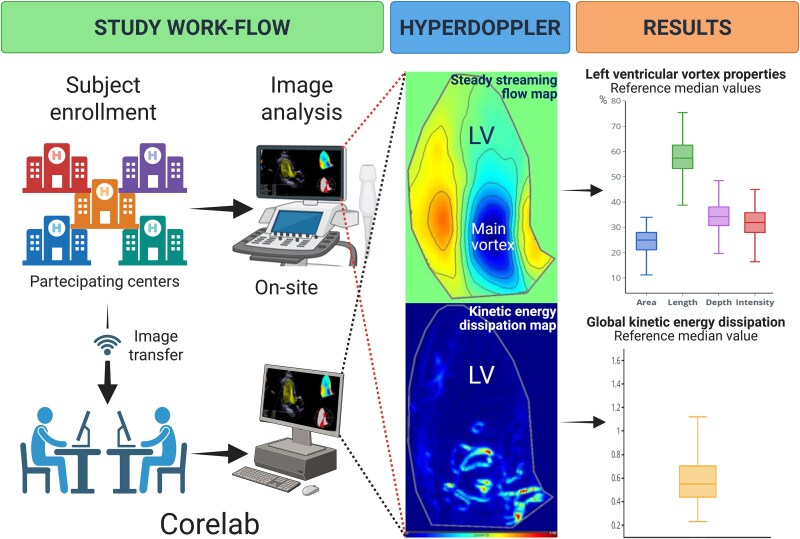
The figure illustrates the study workflow (left), the HyperDoppler maps used for quantitative assessment of left intraventricular flow dynamics (mid-portion) and the graphs with reference median values of left ventricular vortex properties and global kinetic energy dissipation (right).

## Introduction

Assessment of left ventricular (LV) function by analysis of flow dynamics inside the LV chamber has gained increasing interest using both four-dimensional-flow magnetic resonance imaging (4D-flow MRI) and contrast ultrasound techniques, which have been applied especially in patients with ischaemic heart disease, heart failure, and heart valve diseases.^1–5^ However, 4D-flow MRI is not widely accessible and contrast ultrasound is limited by the cost and risks of the contrast agents. In recent years, colour Doppler-based ultrasound techniques have been developed to bring evaluation of intracardiac flow dynamics into clinical practice, including the latest HyperDoppler technique (Esaote, Genova, Italy). This technique (described in Supplementary material) derives from the software HyperFlow, used to analyse intracardiac flow dynamics in conjunction with contrast administration.^6^ HyperDoppler has been applied to athletes and patients with various cardiac diseases.^7–11^ However, although several measures have been utilized to evaluate flow dynamics properties, reference values for these measures have not yet been established, which limits the interpretation of quantitative assessments. Furthermore, there is a lack of data regarding the effects of age, gender, and other physiological and echocardiographic variables. The NORVORTEX (NORmal VORTEX) study was designed to prospectively establish reference values for key HyperDoppler measures, assess reproducibility, examine the effects of age and gender, and determine whether the physiology of left intra-ventricular flow dynamics is accurately captured by quantitative HyperDoppler evaluations.

## Methods

### Study subjects

Thirteen echocardiographic laboratories prospectively enrolled healthy normal subjects and submitted colour Doppler images to an independent core laboratory (corelab) for the HyperDoppler analyses. Inclusion and exclusion criteria are reported in Supplementary material. Subjects were divided into three age groups: 20–39, 40–59, and 60–79 years. The study was approved by the Padova Ethics Committee (ID Study: 21280, CESC Codex: 5601/AO/22, URC Codex: AOP2772). Written informed consent was obtained from participants.

### Image acquisition

#### Standard echocardiographic examination

A comprehensive echocardiographic, Doppler, colour Doppler, and tissue Doppler examination was performed using an echocardiographic scanner equipped with a 1–5 MHz electronic phased-array transducer (MyLab X8, Esaote, Genova, Italy) as described in Supplementary material. Noninvasive blood pressure and heart rate were recorded at the time of the echocardiographic examination using the standard non-invasive approach.

#### HyperDoppler image acquisition

A standard apical colour Doppler long-axis view was acquired during end-expiratory breath-hold in a cineloop format, including one cardiac cycle defined by the R-R interval on the electrocardiographic trace. Depth and sector width were set up to achieve a colour Doppler frame rate of ≥21 fps. Care was taken to include as much as possible of the LV cavity and the LV outflow tract (LVOT) within the colour Doppler sector angle. Colour Doppler pulse repetition frequency was set at 4.4 KHz. Each echo-lab was asked to acquire three different cineloops for on-site measurements. Thereafter, other three different cineloops were acquired with modified echo-scanner configuration in a digital raw-data format (DICOM format), which after anonymization were sent to the corelab in Padova using REDCap (*Research Electronic Data Capture*, Vanderbilt University, Nashville, Tennessee) for central analysis. All image cineloops were acquired attempting to keep heart rate stable across acquisitions

#### HyperDoppler image analysis

HyperDoppler measurements generated by the corelab were used to derive reference values. At the corelab, these measurements were performed on an external workstation by two expert readers, defined as physicians with profound knowledge of the principles and practice of the HyperDoppler technique and publications in the field. The local echo-labs also performed the same evaluations on-board the echo-scanner limited to the subjects examined in each echo-lab. These latter measurements were compared with those provided by the corelab. Readers at each echo-lab were trained for acquisition, analysis, and interpretation of the HyperDoppler images by Esaote specialists before the conduct of the study. At the Padova centre, similarly trained readers (different from corelab readers) performed the HyperDoppler evaluations on-board the echo-scanner as in all the other echo-labs.

### Image retrieving and processing

**Figure 1:**
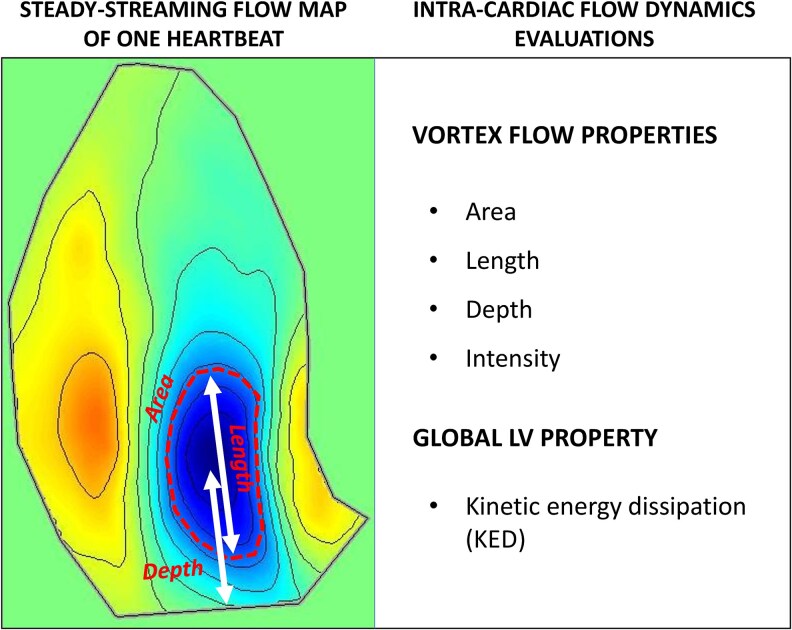
Steady-streaming flow map of one heartbeat, showing blood flow rotating clockwise and counterclockwise. The dotted line schematically circumscribes the compact region of the clockwise vortical flow, where the HyperDoppler geometrical (area, length) and positional (depth) measures are performed by the algorithm. All the HyperDoppler intracardiac flow dynamics evaluations used in the study are listed in the panel on the right. See text for details.

For measurements, both on the external workstation and on-board the echo-scanner, first the stored image cineloops were retrieved. Then, a region of interest was traced on the endocardial borders by the reader to include the LV cavity at end-diastole. The mitral annulus plane was not traced, because it is automatically recognized by the software as the distance between the mitral annulus hinge points. The LVOT was identified using a straight line, traced below (5–10 mm) the aortic valve plane. Finally, the vector velocity recovering algorithm was launched. The automatic output of this algorithm included a steady streaming flow field map (*Figure 1*) and a table with the values of quantitative LV flow dynamics measures (all described below). For each HyperDoppler measure, the average value of measurements performed on the three acquired image cineloops was taken. Software version F13 was used on-board the echo-scanner and 2.01 on the external workstation.

### Quantitative flow dynamics measures

Scalar dimensionless measures related to the LV flow dynamics have been previously described.^6,7^ In brief, to obtain measures characterizing the geometry and position of the main vortex within the LV, the steady streaming (heartbeat average) flow field was computed by the software to evaluate the overall circulatory pattern in the LV during one heartbeat (*Figure 1*). The intraventricular vortex was defined as the compact region about the steady streaming vortex centre, where the streamfunction was larger than one half of its peak value at the vortex centre. The vortex properties of this net circulatory region were expressed by the following measures (*Figure 1*): (i) the vortex area, normalized with the LV area (in %), (ii) the vortex length along the base-apex direction, normalized with the LV length (in %), (iii) the vortex depth (i.e. the distance of the vortex centre from the LV base), normalized with the LV length (in %), and (iv) the vortex intensity (i.e. the integral of the vorticity inside the vortex), normalized with the total LV vorticity (in %). The vortex intensity (sometime called vortex circulation) was expressed as a negative number if vortex rotation was clockwise and as a positive number if counterclockwise. On the steady streaming flow map, clockwise vortex rotation was depicted in blue and counterclockwise vortex rotation in red (*Figure 1*). The energetic properties of LV flow were quantified using kinetic energy dissipation (KED), with global KED (gKED) representing the total amount of kinetic energy dissipated within the LV chamber due to viscous friction over the cardiac cycle.^6,7^ The gKED was considered as the value integrated over the entire LV, normalized with the average kinetic energy to avoid direct dependence on the LV size.^6,7^ This is a distinct definition compared to all the other flow dynamics measures, which refer only to the main vortex inside the LV.

### Statistical analysis

The normal distribution was tested for each variable with the Kolmogorov–Smirnov test. Since most variables had a non-normal distribution, descriptive statistics was reported as medians with the first (Q1) and third (Q3) quartiles for continuous variables and as absolute numbers and percentages for categorical variables. As a measure of dispersion expressed in %, the quartile coefficient of dispersion (QCD) was also calculated as the Q3—Q1 divided by the sum of Q3 + Q1. The Mann–Whitney and Kruskal–Wallis tests were performed to compare continuous variables, when appropriate. The chi-squared test was performed to compare categorical variables. Correlations were assessed using the Spearman’s rank correlation coefficient (Rho). A weak correlation was defined as a correlation coefficient <0.3, whereas a strong correlation was defined as a correlation coefficient >0.7. Statistical analysis was performed on the database obtained after outliers’ elimination for every HyperDoppler measure. Outlier detection was performed using the interquartile range (IQR: Q3-Q1). Precisely, an outlier was defined as any data point <Q1—(1.5 IQR) or > Q3+ (1.5 IQR). For the HyperDoppler measures only, the mean ± standard deviation and coefficient of dispersion were also reported for better comparison with previous studies. To test the corelab reproducibility of the HyperDoppler measures, the two corelab readers independently repeated the evaluation in 32 randomly extracted colour Doppler cineloops, without knowledge of the previous measurements. Reproducibility was expressed calculating the intraclass correlation coefficient together with the 95% confidence interval; linear regression and Bland–Altman analyses were also performed. A *P* < 0.05 was considered statistically significant. Analyses were performed using the Medcalc software, Ostend, Belgium, v. 23.2.1.

## Results

**Figure 2:**
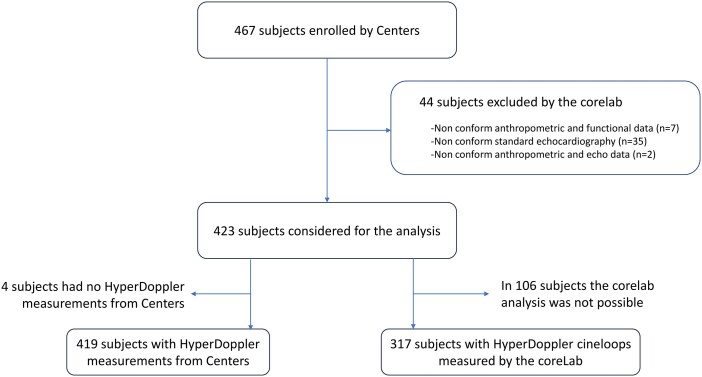
Study flow chart.

The 13 echo-labs enrolled 467 subjects. Of these, 44 subjects were excluded because of non-conformity to study protocol (see Supplementary data online, Table  *S1*) and the remaining 423 subjects formed the study group (*Figure 2*). In 419 subjects the HyperDoppler analysis was performed on-site, and results were sent to the corelab; for the remaining four subjects, the corelab received only the standard echocardiographic measurements, not the HyperDoppler measurements. In 317 subjects, the corelab could perform the HyperDoppler analysis on the colour Doppler cineloops. The reasons for the failure to evaluate colour Doppler images in 106 subjects at the corelab were related to study procedural or technical problems (see Supplementary data online, Table  *S2*), not to the feasibility of the HyperDoppler technique.

### Study subjects’ characteristics

The main characteristics of all the 423 study subjects and the 317 subjects with the HyperDoppler evaluation at the corelab are reported in *Table 1*. No difference was found between these two groups, thus excluding a selection bias. The complete set of echocardiographic measures for all study participants is provided in Supplementary data online, Table  *S3*.

**Table 1:** Main characteristics of study subjects

	Overall subjects			Males			Females			
	*N*	Median	25th–75th Percentile	*N*	Median	25th–75th Percentile	N	Median	25th–75th Percentile	*P* value*
Age (years)	423	36.0	28.0–55.0	223	35.0	28.0–52.0	200	37.0	28.0–57.5	0.268
Height (cm)	423	170.0	165.0–179.0	223	178.0	173.0–181.0	200	165.0	160.0–170.0	<0.001
Weight (Kg)	423	70.0	60.0–77.0	223	75.0	70.0–82.0	200	60.0	55.0–65.0	<0.001
BSA (m^2^)	423	1.81	1.66–1.95	223	1.93	1.84–2.02	200	1.66	1.58–1.74	<0.001
BMI	423	23.0	21.1–25.1	223	24.1	22.2–25.8	200	22.0	20.0–23.5	<0.001
Heart rate (bpm)	423	68.0	62.0–74.0	223	68.0	62.0–74.0	200	68.5	62.0–75.0	0.247
SBP (mmHg)	423	120.0	110.0–125.0	223	120.0	110.0–125.0	200	120.0	110.0–125.0	0.505
DBP (mmHg)	423	70.0	70.0–80.0	223	70.0	70.0–80.0	200	70.0	67.0–80.0	0.425
LV EDD index (mm/m^2^)	423	25.2	23.4–27.1	223	24.5	23.0–26.0	200	26.3	24.1–28.0	<0.001
LV ejection fraction (%)	423	63.0	60.0–67.0	223	63.0	60.0–66.0	200	64.0	61.0–68.0	0.004

	Subjects evaluated at the corelab			Males			Females			
	*N*	Median^a^	25th–75th Percentile	*N*	Median^a^	25th–75th Percentile	*N*	Median^a^	25th–75th Percentile	*P* value*
Age (years)	317	34.0	28.0–53.0	166	31.5	28.0–49.0	151	37.0	28.0–57.0	0.050
Height (cm)	317	170.0	165.0–179.0	166	178.0	173.0–182.0	151	165.0	161.0–170.0	<0.001
Weight (Kg)	317	68.0	60.0–76.0	166	75.0	70.0–82.0	151	60.0	55.0–64.0	<0.001
BSA (m^2^)	317	1.80	1.65–1.94	166	1.93	1.85–2.01	151	1.65	1.58–1.74	<0.001
BMI	317	22.9	21.0–24.7	166	23.8	22.1–25.7	151	21.6	20.0–23.2	<0.001
Heart rate (bpm)	317	70.0	61.0–75.0	166	69.0	60.0–74.0	151	70.0	62.0–75.8	0.121
SBP (mmHg)	317	120.0	110.0–125.0	166	120.0	110.0–125.0	151	120.0	110.0–125.0	0.883
DBP (mmHg)	317	70.0	70.0–80.0	166	70.0	70.0–80.0	151	70.0	68.0–80.0	0.134
LV EDD index (mm/m^2^)	317	25.4	23.6–27.1	166	24.7	23.3–25.8	151	26.4	24.3–28.2	<0.001
LV ejection fraction (%)	317	64.0	61.0–68.0	166	64.0	60.0–67.0	151	64.0	61.0–68.8	0.032

BMI, body mass index; BSA, body surface area; DBP, diastolic blood pressure; EDD, end-diastolic diameter; LV, left ventricle; SBP, systolic blood pressure.

^a^No statistically significant difference was found between median values of subjects evaluated at the corelab and the overall groups of study subjects.

^*^Comparison between males and females.

### Corelab evaluations

**Figure 3:**
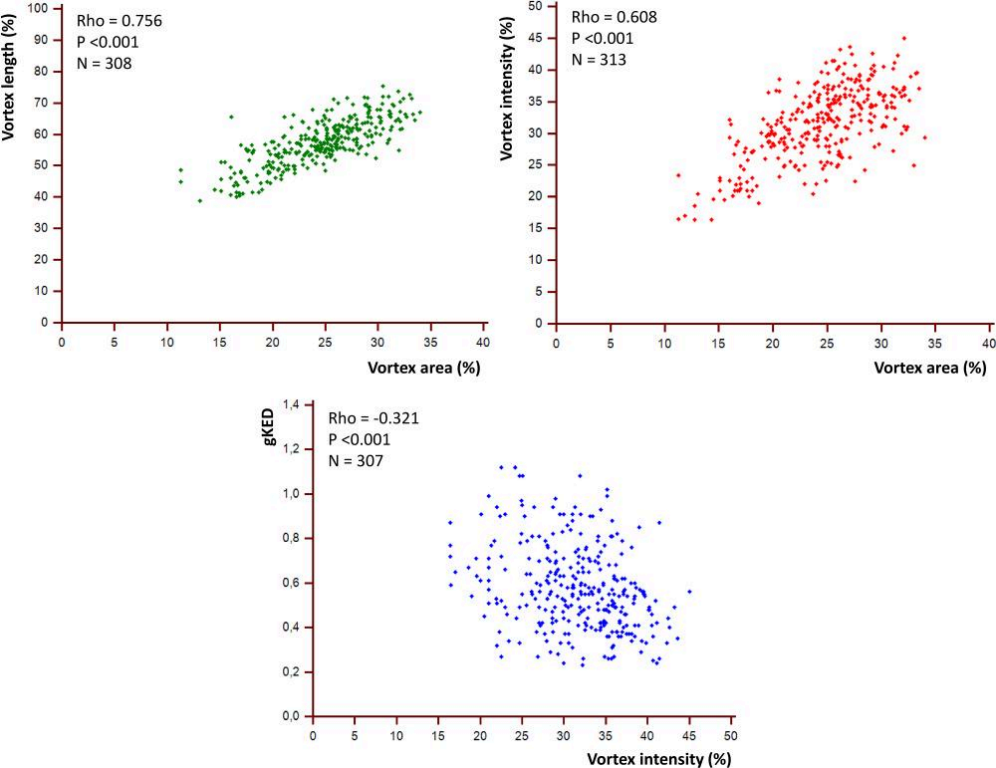
Significant correlations among the HyperDoppler measures, shown through relative scattergrams. Intensity is expressed as absolute values. gKED: global kinetic energy dissipation.

The number of outlier values for the HyperDoppler measures is reported in Supplementary data online, Table  *S4*, while the study values are shown graphically in the Graphical abstract and reported in *Table 2* as medians and Table  *S5* as means. The slightly lower number of subjects in *Tables 2* and *S5*, relative to the total of 317 subjects, is due to the exclusion of the outlier values. Correlations among the various HyperDoppler measures are reported in *Table 3* and shown in *Figure 3*; intensity values were converted to absolute values because all were negative. Measurements relative to single echo-labs are shown in Supplementary data online, Tables  *S6*–*S10* and Supplementary data online, *F*igures  *S1*–*S5*. All these tables and figures do not include the initially excluded outlier values.

**Table 2:** Comparison of HyperDoppler measures between patients evaluated at the corelab and on-site

	N	Median	25th–75th Percentile	*P* value*	QCD	*P* value**
Corelab	317					
Vortex area	315 (99.4%)	25.0%	21.1–28.0%	—	14.1%	—
Vortex length	309 (97.5%)	57.4%	53.3–62.6%	—	8.0%	—
Vortex depth	315 (99.4%)	34.3%	30.7–38.1%	—	10.8%	—
Vortex intensity	315 (99.4%)	−31.9%	−28.0–−35.8%	—	12.2%	—
gKED	309 (97.5%)	0.55	0.44–0.70	—	22.8%	—
On-site—Matched subjects	317					
Vortex area	310 (97.8%)	24.2%	20.7–27.5%	0.087	14.1%	0.995
Vortex length	305 (96.2%)	56.5%	50.3–63.0%	0.249	11.2%	0.457
Vortex depth	308 (97.2%)	33.2%	28.7–38.2%	0.035	14.2%	0.592
Vortex intensity	312 (98.4%)	−28.4%	−23.1–−32.6%	<0.001	17.1%	0.518
gKED	305 (96.2%)	0.59	0.47–0.80	0.007	26.0%	0.964
On-site—All subjects	419					
Vortex area	414 (98.8%)	24.2%	20.6–28.0%	0.303	15.2%	0.846
Vortex length	406 (96.9%)	57.4%	50.8–63.7%	0.591	11.3%	0.347
Vortex depth	405 (96.7%)	33.2%	28.7–38.1%	0.022	14.1%	0.402
Vortex intensity	416 (99.3%)	−28.7%	−23.7–−32.8%	<0.001	16.1%	0.429
gKED	403 (96.2%)	0.61	0.47–0.80	<0.001	26.0%	0.623

The different number of patients for each HyperDoppler measure depends on the number of the initially excluded outlier values

gKED, global kinetic energy dissipation; QCD, quartile coefficient of dispersion.

^*^Medians of on-site vs. corelab evaluations.

^**^QCDs of on-site vs. corelab evaluations.

**Table 3:** Correlations between the corelab HyperDoppler measures

	Vortex area	Vortex length	Vortex depth	Vortex intensity	gKED
Vortex area	*N* = 315	—	—	—	—
Vortex length	Rho = 0.756	—	—	—	—
	*N* = 308	*N* = 309	—	—	—
	*P* < 0.001	—	—	—	—
Vortex depth	Rho = 0.194	Rho = 0.255	—	—	—
	*N* = 313	*N* = 308	*N* = 315	—	—
	*P* < 0.001	*P* < 0.001	—	—	—
Vortex intensity	Rho = 0.608	Rho = 0.408	Rho = −0.057	—	—
	*N* = 313	*N* = 308	*N* = 313	*N* = 315	—
	*P* < 0.001	*P* < 0.001	*P* = 0.316	—	—
gKED	Rho = −0.070	Rho = −0.037	Rho = 0.081	Rho = −0.321	—
	*N* = 307	*N* = 301	*N* = 307	*N* = 307	*N* = 309
	*P* = 0.220	*P* = 0.526	*P* = 0.156	*P* < 0.001	—

gKED, global kinetic energy dissipation.

#### Effect of age

The HyperDoppler measures in the three age groups are presented in *Table 4* and Supplementary data online, *F*igures  *S6*–*S10*. Only vortex length median values showed a slight statistically significant increase with age, especially in older women. However, the correlation between age and vortex length was negligible, albeit statistically significant (*Table 5*).

**Table 4:** HyperDoppler corelab measures in age groups

	Group 20–39 years		Group 40–59 years		Group 60–79 years		
	*N*	Median (25th–75th percentile)	*N*	Median (25th–75th percentile)	*N*	Median (25th–75th percentile)	*P* value
All subjects							
Vortex area (%)	187	24.9 (20.8–27.6)	77	25.0 (21.0–26.8)	51	25.1 (22.7–30.3)	0.206
Vortex length (%)	184	56.5 (52.8–62.3)	77	56.7 (50.9–61.6)	48	61.3 (56.5–65.2)	0.005
Vortex depth (%)	185	33.8 (30.7–37.9)	79	34.8 (30.7–38.1)	51	35.7 (31.2–39.4)	0.291
Vortex intensity (%)	185	−32.1 (−28.4–−36.1)	79	−30.6 (−27.1–−34.8)	51	−32.5 (−27.0–−36.0)	0.159
gKED	181	0.54 (0.42–0.70)	78	0.59 (0.49–0.71)	50	0.55 (0.38–0.65)	0.259
Males							
Vortex area (%)	107	24.9 (21.2–27.1)	37	25.0 (20.7–27.3)	21	25.1 (19.1–30.0)	0.879
Vortex length (%)	107	57.2 (54.3–62.3)	38	56.2 (51.0–61.6)	19	61.2 (56.5–64.5)	0.097
Vortex depth (%)	107	32.8 (29.5–36.5)	38	34.5 (29.0–37.2)	21	32.2 (29.1–35.4)	0.709
Vortex intensity (%)	107	−32.5 (−28.6–−36.0)	38	−31.1 (−27.0–−34.7)	21	−33.0 (−25.6–−36.6)	0.253
gKED	104	0.52 (0.41–0.67)	38	0.59 (0.41–0.67)	21	0.56 (0.38–0.65)	0.442
Females							
Vortex area (%)	80	24.6 (20.2–28.4)	40	25.1 (21.4–26.7)	30	25.1 (22.9–31.2)	0.141
Vortex length (%)	77	55.7 (49.8–62.3)	39	57.3 (51.2–61.5)	29	61.3 (57.3–65.7)	0.028
Vortex depth (%)	78	35.7 (31.9–39.1)	41	35.2 (31.6–38.3)	30	36.7 (34.9–41.3)	0.090
Vortex intensity (%)	78	−31.9 (−28.3–−36.2)	41	−30.4 (−27.8–−35.0)	30	−32.2 (−29.1–−35.4)	0.572
gKED	77	0.57 (0.48–0.73)	40	0.62 (0.51–0.72)	29	0.55 (0.40–0.64)	0.434

gKED: global kinetic energy dissipation.

**Table 5:** Correlations between HyperDoppler measures and age, sex, and echocardiographic variables

	Vortex area	Vortex length	Vortex depth	Vortex intensity	gKED
Age	Rho = 0.103	Rho = 0.117	Rho = 0.064	Rho = −0.016	Rho = 0.025
	*N* = 315	*N* = 309	*N* = 315	*N* = 315	*N* = 309
	*P* = 0.069	*P* = 0.039	*P* = 0.256	*P* = 0.782	*P* = 0.666
Gender	Rho = 0.029	Rho = −0.035	Rho = 0.211	Rho = −0.030	Rho = 0.129
	*N* = 315	*N* = 309	*N* = 315	*N* = 315	*N* = 309
	*P* = 0.611	*P* = 0.544	*P* < 0.001	*P* = 0.598	*P* = 0.023
LV EDV	Rho = −0.030	Rho = 0.002	Rho = −0.079	Rho = −0.061	Rho = −0.009
	*N* = 315	*N* = 309	*N* = 315	*N* = 315	*N* = 309
	*P* = 0.596	*P* = 0.966	*P* = 0.164	*P* = 0.277	*P* = 0.878
LV ED Length	Rho = −0.017	Rho = −0.077	Rho = −0.175	Rho = −0.005	Rho = −0.010
	*N* = 310	*N* = 303	*N* = 309	*N* = 309	*N* = 303
	*P* = 0.771	*P* = 0.182	*P* = 0.002	*P* = 0.930	*P* = 0.869
LV ejection fraction	Rho = 0.060	Rho = 0.093	Rho = 0.124	Rho = −0.095	Rho = 0.156
	N = 315	*N* = 309	N = 315	*N* = 315	*N* = 309
	*P* = 0.289	*P* = 0.105	*P* = 0.028	*P* = 0.091	*P* = 0.006
Aortic TVI	Rho = 0.046	Rho = 0.094	Rho = 0.210	Rho = −0.150	Rho = 0.136
	*N* = 309	*N* = 303	*N* = 309	*N* = 309	*N* = 303
	*P* = 0.416	*P* = 0.102	*P* < 0.001	*P* = 0.008	*P* = 0.018
Peak E wave	Rho = −0.038	Rho = −0.080	Rho = 0.160	Rho = −0.011	Rho = 0.149
	*N* = 312	*N* = 306	*N* = 312	*N* = 312	*N* = 306
	*P* = 0.499	*P* = 0.165	*P* = 0.005	*P* = 0.847	*P* = 0.009
Mitral TVI	Rho = 0.000	Rho = 0.017	Rho = 0.197	Rho = −0.035	Rho = 0.143
	*N* = 306	*N* = 300	*N* = 306	*N* = 306	*N* = 300
	*P* = 0.993	*P* = 0.763	*P* = 0.001	*P* = 0.548	*P* = 0.013

ED, end-diastolic; EDV, end-diastolic volume; gKED, global kinetic energy dissipation; LV, left ventricle; TVI, time velocity integral.

#### Effect of gender

HyperDoppler measures for the two gender groups are presented in *Table 6*. Median values of vortex depth and gKED were higher in women, with only a minimal difference observed for gKED (see Supplementary data online, *F*igure  *S11*). For both measures, correlations with gender were weak to very weak in magnitude, although they were statistically significant (*Table 5*).

**Table 6:** HyperDoppler corelab measures in gender groups

	Males			Females			
	*N*	Median	25th–75th percentile	*N*	Median	25th–75th percentile	*P* value
Vortex area (%)	165	24.9	21.1–27.5	150	25.1	21.0–28.1	0.611
Vortex length (%)	164	57.4	53.9–62.3	145	57.3	51.2–62.9	0.543
Vortex depth (%)	166	33.0	29.2–36.9	149	36.0	31.9–39.1	<0.001
Vortex intensity (%)	166	−32.1	−27.9–−35.9	149	−31.4	−28.1–−35.8	0.598
gKED	163	0.55	0.41–0.67	146	0.56	0.48–0.71	0.023

gKED, global kinetic energy dissipation.

#### Effect of echocardiographic parameters

Correlations between HyperDoppler and echocardiographic measures are shown in *Table 5*. Only weak or very weak, though statistically significant, correlations were observed between selected HyperDoppler measures (vortex depth, vortex intensity, and gKED) and LV end-diastolic length, ejection fraction, aortic TVI, peak E wave, and mitral TVI.

### On-site evaluations

The number of outlier values for the HyperDoppler measures is reported in Supplementary data online, Table  *S5*, while the study values are shown in *Table 2* as medians and Table  *S5* as means. As for the corelab evaluations, the slightly lower number of subjects in *Tables 2* and *S5*, relative to the total of 419 subjects, is due to the exclusion of the outlier values. Compared to the corelab evaluations, on-site median values of all subjects were similar for vortex area and length, lower for vortex depth and intensity, and higher for gKED (*Table 2*). On-site QCD values were generally slightly higher compared to the corelab, albeit statistically insignificant (*Table 2*). Considering the subgroup of subjects evaluated on-site who were matched with those analysed by the corelab, results were similar (*Table 2*). Correlations between the on-site and corelab HyperDoppler measures were as follows: vortex area, Rho = 0.548 (*N* = 308; *P* < 0.001); vortex length, Rho = 0.576 (*N* = 297; *P* < 0.001); vortex depth, Rho = 0.459 (*N* = 306; *P* < 0.001); vortex intensity, Rho = 0.592 (*N* = 310; *P* < 0.001); and gKED, Rho = 0.718 (*N* = 301; *P* < 0.001). Measurements relative to the single echo-labs are shown in Supplementary data online, Tables  *S6*–*S10* and Supplementary data online, *F*igures  *S1*–S5. *Tables 2*, *S6*–*S10* and Supplementary data online, *F*igures  *S1*–*S5* do not include the initially excluded outlier values.

### HyperDoppler reproducibility

The ICC values were excellent (≥0.950) for each HyperDoppler measure (see Supplementary data online, Table  *S11*). At the Bland–Altman analysis, bias was always <5% of the mean value (see Supplementary data online, *F*igures  *S12*–*S16*).

## Discussion

Results of the present study demonstrate that the HyperDoppler technique is robust and reproducible, particularly for the assessment of LV vortex properties, which are characterized by a smaller variability compared to gKED and are not significantly influenced by age or sex. Furthermore, quantitative HyperDoppler measures effectively characterize left intraventricular flow dynamics in a physiologically coherent manner. Collectively, these findings support the clinical applicability of HyperDoppler and extend previous reports, which were limited by small groups of normal subjects, heterogeneous definitions of normality, lack of data on the influence of physiological variables, and single- or dual-centre study design.^7–11^

### Characteristic features of the HyperDoppler measures

The HyperDoppler measures of vortex properties are derived from the steady streaming flow field, while gKED is calculated as a time integral over the full heartbeat. As a result, all measures are relative to the entire cardiac cycle, rather than specific time points within the heartbeat. This holistic approach is crucial because associating flow properties with individual time intervals can be complex. Blood flow at any given moment is influenced by events that occurred earlier in the cycle. For example, the flow at the onset of systole is influenced by vortices generated during the preceding diastole. The HyperDoppler technique stands out by providing a comprehensive overview of LV flow dynamics throughout the entire cardiac cycle.

### Description of LV flow dynamics physiology

Based on median values, the LV vortex area was approximately one quarter of the LV cavity, while vortex length accounted for nearly 60% of the LV longitudinal dimension in healthy hearts. The main vortex was typically located closer to the LV base than the apex, with its centre positioned at approximately 35% of the LV cavity length. LV vortex intensity was consistently negative, indicating a clockwise rotation of the primary LV vortex in the long-axis view. Median LV vortex intensity represented approximately one third of the total LV vorticity. Overall, these findings confirm that in healthy hearts the LV blood flow observed in the apical long-axis view is organized into a stable, clockwise-rotating main vortex with consistent size and spatial distribution. The median LV gKED value was 0.55, indicative of efficient intraventricular flow organization. However, this parameter exhibited the greatest variability among all measured metrics (*Table 2*, Supplementary data online, Table  *S5*).

### Correlations between the HyperDoppler measures

Several considerations are forthcoming from the correlations between the HyperDoppler measures (*Table 3*, *Figure 3*). First, vortex area and length were strongly correlated to each other (Rho = 0.756, *P* < 0.001). This finding reflects the elongated geometry of the normal LV vortex along the longitudinal axis. Second, both vortex area and length showed a very weak or negligible correlation with vortex depth. This finding suggests that in normal subjects, the position of the main vortex within the LV is relatively independent on vortex size, especially on vortex area. Third, vortex intensity moderately correlated with vortex area (Rho = 0.608, *P* < 0.001). This was expected, as the vortex intensity partly depends on vortex size, in addition to vorticity (specifically, vortex intensity is the product of vortex area and vorticity). Fourth, gKED showed no correlation with the geometrical or positional properties of the vortex; however, a weak-to-moderate inverse correlation was observed between gKED and vortex intensity, expressed as an absolute value (Rho = −0.321, *P* < 0.001). This inverse correlation may be explained considering that the main LV vortex tends to preserve kinetic energy, reducing KED. On the other hand, gKED represents KED throughout the LV cavity, not only in the main LV vortex. All the observations above are coherent with physiology of LV flow dynamics evaluated using three-dimensional MRI^12^ and contrast-based ultrasound techniques.^6^

### Effect of age and gender

Most HyperDoppler measures did not vary with age; however, vortex length showed a statistically significant increase, particularly in the older age group (*Table 4*). The relevance of this increase is limited by the substantial overlap in vortex length values across the three age groups (see Supplementary data online, *F*igure  *S7*) and by the very weak—though statistically significant—correlation between age and vortex length (*Table 5*).

There were no differences in the median values of vortex area, length, or intensity between men and women, whereas median vortex depth and gKED values were higher in women (*Table 6*). However, the difference in gKED was minimal, and for both measures, there was substantial overlap in values between the two gender groups (see Supplementary data online, *Figure S11*), along with weak to very weak—albeit statistically significant—correlations with gender (*Table 5*). Accordingly, despite reaching statistical significance, differences in vortex depth and gKED are likely to have limited practical relevance.

### Effect of echocardiographic variables

The size of the LV, expressed as end-diastolic volume, did not correlate with any HyperDoppler measure (*Table 5*). This was expected, as all the HyperDoppler measures were indexed to account for the LV dimensions, with the aim to define parameters describing flow properties only. Vortex depth demonstrated a weak inverse correlation with LV end-diastolic length (*Table 5*).

In our normal subjects, geometrical vortex properties (area and length) did not correlate with standard echocardiographic measures of LV function (*Table 5*), whereas only weak or very weak—though statistically significant—correlations were observed for vortex depth, vortex intensity, and gKED (*Table 5*). The lack of strong correlations between HyperDoppler measures and conventional indices of LV systolic and diastolic function may reflect the limited range of values observed in normal subjects. These relationships could differ when both healthy individuals and patients with cardiac pathology are considered, as a broader spectrum of HyperDoppler values would then be captured. Indeed, when the contrast-based HyperFlow technique was applied to healthy subjects and patients with myocardial infarction and LV systolic dysfunction, moderate linear correlations were observed between gKED and LV ejection fraction (*r* = 0.57) and global longitudinal strain (r = −0.61), whereas no significant associations were identified with vortex-related parameters.^13^ The observed correlations may also partly reflect the potential incremental value of intracardiac flow dynamics metrics, which provide complementary information by characterizing flow throughout the entire cardiac cycle.

### Comparison with on-site evaluations

On-site median values of LV vortex area and length were similar to those obtained by the corelab, while vortex depth, intensity, and gKED median values statistically differed (*Table 2*). Correlations between the on-site and corelab evaluations were predominantly moderate, ranging from 0.459 to 0.718. These findings suggest that, in addition to the initial training provided by the vendor’s specialists, a learning curve is probably needed to perform measurements optimally. It should be also considered that on-site and corelab measurements were not performed on the same colour Doppler cineloops, although ultrasound images were acquired sequentially at each echo-lab.

### Results in individual echo-labs

For each HyperDoppler measure, there was a difference in the HyperDoppler evaluations performed in the different echo-labs (see Supplementary data online, Tables  *S6*–*S10*, Supplementary data online, *F*igures  *S1*–*S5*). Interestingly, these differences persisted at the corelab evaluation. Variations observed across different echo-labs are likely related to the fact that the vortex properties of subjects evaluated at each echo-lab were not perfectly overlapping, and the number of examined subjects varied.

### Potential applications and perspectives

One of the most important applications of HyperDoppler is the assessment of LV function. By enabling quantitative characterization of intraventricular vortex dynamics and KED, HyperDoppler extends functional evaluation beyond conventional indices such as LV ejection fraction or regional wall-motion analysis, which may be insensitive to early or subtle dysfunction. Quantitative assessment of intraventricular flow dynamics supports a conceptual shift in LV evaluation, framing LV dysfunction not only as impaired myocardial contractility and/or relaxation, but also as a disorder of flow efficiency.

In heart failure with reduced ejection fraction, LV dilatation and mechanical dyssynchrony disrupt normal intraventricular flow organization, impairing the transfer of kinetic energy from diastolic filling to systolic ejection. In some cases, a large and persistent LV vortex develops that retains kinetic energy but is malpositioned relative to the LV outflow tract—typically displaced towards the apex—thereby limiting effective energy redirection and resulting in reduced KED.^4,7^ Conversely, increased KED may be observed in the presence of turbulent flow, vortex fragmentation, and elevated LV filling pressures.^4,14^ Collectively, these findings suggest that HyperDoppler may help distinguish distinct haemodynamic and remodelling phenotypes within heart failure, complementing conventional functional measures.

Beyond phenotyping, HyperDoppler offers potential utility for monitoring changes in LV flow dynamics following therapeutic interventions, such as correction of valvular disease or relief of outflow tract obstruction in hypertrophic cardiomyopathy.^3,15^ Integration of HyperDoppler-derived flow metrics with advanced functional parameters—including myocardial work, LV efficiency, ventricular–arterial coupling, and maximal LV elastance—may further enhance its translational relevance. However, longitudinal data linking HyperDoppler measures to clinical outcomes remain limited, underscoring the need for prospective studies to define their prognostic value and role in patient management.

### Study limitations

The study group included normal subjects who were Caucasian. This may limit generalizability of results to individuals of non-Caucasian origin. Even though we made significant efforts to ensure the normalcy of the study group, long-term follow-up and natriuretic peptides were not part of the protocol. The number of normal subjects >70 years old at the corelab analysis was small (*n* = 6). This is related to the known difficulty to find normal subjects with the oldest age and suggest caution in interpreting results in this age group. This study was performed with subjects at rest. The effects of exercise and preload and afterload variations on HyperDoppler measures remain unassessed, as does the influence of heart rates >100 bpm or <40 bpm. The relatively high QCD values of the LV gKED should warrant caution when interpreting results obtained with this parameter. Although a direct comparison between HyperDoppler and 4D-flow MRI in the same normal subjects is lacking, reconstruction of the two-dimensional vector flow field from one-dimensional colour Doppler velocity data has been previously validated.^16^ However, the HyperDoppler technique itself has not yet been validated in patients with diverse pathological LV morphologies and phenotypes. The HyperDoppler technique is currently semi-automated, with a fully automated version expected to streamline the measurement process. Although age did not significantly influence HyperDoppler measures in this study, age-related variations may still occur in flow dynamics measures taken at specific points within the cardiac cycle, such as during early LV filling.^5^ HyperDoppler reproducibility could be different in patients with varying cardiac pathology and sub-optimal image quality, not only due to inadequate acoustic windows but also as a result of imprecise or inaccurate image acquisition. Accounting for varying image quality is essential when performing HyperDoppler analysis in clinical practice. Finally, the presence of valvular diseases can influence intraventricular flow patterns, as shown with other techniques.^3^ However, in our study cohort, only healthy subjects were included.

### Conclusions

Reference values for left intraventricular flow dynamics were established using HyperDoppler, a reproducible technique that enables the assessment of intracardiac flow dynamics in clinical practice.

## Supplementary Material

qyag050_Supplementary_Data

## Data Availability

The data underlying this article will be shared on reasonable request to the corresponding author.

## Lead author biography

Donato Mele, MD. Former Associate Professor of Cardiology; currently Senior Scholar, Department of Cardiac, Thoracic, and Vascular Sciences and Public Health, University of Padova, Italy. Head of the Cardiology Unit, Ravenna33 Clinic, Ravenna, Italy.
