# Supplementary material for: Evaluation of left intraventricular flow dynamics using the novel non-contrast HyperDoppler technique
Source: Eur Heart J Imaging Methods Pract. 2026 Mar 19;4(1):qyag050. doi: 10.1093/ehjimp/qyag050 (PMC13137989; doi:10.1093/ehjimp/qyag050)
Supplement: qyag050_Supplementary_Data [file qyag050_supplementary_data.pdf]

Evaluation of Left Intraventricular Flow Dynamics Using the Novel Non-contrast HyperDoppler Technique

Running title: HyperDoppler for Vortex Analysis

Riccardo Beccari, MD, Antonella Cecchetto, MD, Vittorio Smarrazzo, MD, Vittoria Miano, MD, Mauro Pepi, MD, Fabio Fazzari, MD, Laura Stefani, MD, Marco Corsi, MD, Matteo Cameli, MD, Marta Focardi, MD, PhD, Concetta Zito, MD, Giancarlo Trimarchi, MD, Jaroslaw Kasprzak, MD, Dominika Filipiak-Strzecka, MD, Chiara Cogliati, MD, Daniela Torzillo, MD, Iolanda Aquila, MD, Ettore Ventura, MD, Jolanda Sabatino, MD, Rosalba De Sarro, MD, Marco Pepe, MD, Pierluigi Incarnato, TFCPC, Giampaolo Bezante, MD, Paolo Costa, MD, Omar Prieto, MD, Marco Maglione, MSc, Raphael Rascón-Sabido, MD, Dario Gregori, PhD, Jagat Narula, MD, Gianni Pedrizzetti, PhD, and Donato Mele, MD

SUPPLEMENTARY MATERIAL

METHODS

Description of the HyperDoppler technique

Consider a two-dimensional (2D) image scan plane where the echocardiographic scanner reports the Doppler velocity at each point X of the plane $V_D(X)$. The velocity is given at each frame time of the acquisition, and at least one image is necessary. The point X coordinates can be expressed in general as the two Cartesian coordinates X = (x,y) or the polar coordinates X = (r,θ), where the radius r is the distance from the focus and θ is the sector angle, transversal to the radial direction.

The initial objective is the evaluation of the 2D velocity vector field V(X) in terms of Cartesian components $V_x(X)$, $V_y(X)$, or polar components $V_r(X)$, $V_\theta(X)$. The polar coordinates are known by Doppler such that the radial velocity is simply $V_r = -V_D$, and the transversal velocity V_θ has to be computed. To this aim, the assumption of flow incompressibility on the plane is made, and the transversal coordinate can be computed by using the planar continuity equation, that in polar coordinates reads:

$$\frac{\partial r V_r}{\partial r} + \frac{\partial V_\vartheta}{\partial \vartheta} = 0 \qquad \text{(A1)}$$

Given that the radial velocity is known by the Doppler measurement in the echocardiographic sector ranging from θ_1 to θ_2, the first term in the continuity Equation (A1) can be computed, and the radial velocity is then evaluated using Equation (A2) by integration:

$$V_\vartheta(r,\vartheta) = V_\vartheta(r,\vartheta_0) - \int_{\vartheta_0}^{\vartheta} \frac{\partial r V_r}{\partial r} d\vartheta \qquad \text{(A2)}$$

where the integration can start from any position, here indicated by θ_0, where the value of the transversal velocity is known. The integration Equation (A2) gives the solution apart from an undetermined function of the radial coordinate V_θ (r, θ_0) and of time that must be specified with

some assumption. Several methods propose different solutions to this problem. The present approach assumes that the velocity field is given by the Doppler component $V_D(X)$ plus an irrotational flow: $V(X) = V_D(X) + U(X)$, where the Doppler part is formally expressed as a vector built by the Doppler component only. The additional irrotational flow, U(X), can be expressed as the gradient of a scalar potential φ as:

$$U(X) = \nabla\phi \qquad \text{(A3)}$$

The choice of an irrotational flow, sometimes called potential flow, is due to the following reasons. A potential flow is the least disturbing flow in the sense that it does not modify the distribution of vorticity, which is the key quantity in any fluid flow; therefore, it does not significantly alter energy dissipation and shear stress distribution in the flow field. A potential flow does not enter, besides rigid transport terms, into the fluid dynamics energy balance. Nevertheless, the irrotational flow permits adjusting mass conservation; it can rather act on mass conservation only. Therefore, the choice of using an irrotational flow as a correction for continuity is the most natural choice from the fluid dynamics principle.

The potential flow permits to satisfy the continuity simply by applying the continuity equation that, in general, is expressed as the constraint of velocity having zero divergence:

$$\nabla \cdot V = 0 \qquad \text{(A4)}$$

that is equivalent to Equation (A1). The Doppler flow alone does not satisfy the continuity Equation (A4) and produces a spatial distribution of divergence. Application of the continuity Equation (A4) to the complete (Doppler plus potential) flow gives the elliptic equation of Poisson type:

$$\nabla^2\phi = -\nabla \cdot V_D \qquad \text{(A5)}$$

where the right-hand side is a known term, computed from the Doppler velocity, and the potential φ is the unknown. Once the potential φ is obtained from the solution of Equation (A5), the total velocity can be computed by Equation (A3).

The elliptic Equation (A5) permits inserting any boundary condition that is physically consistent. It gives exactly the irrotational flow that is physically required to fulfill the continuity constraint and the desired boundary conditions.

The solution is here obtained in the Cartesian image coordinates. These allow the development calculation techniques that are particularly efficient and also ensure a more uniform distribution of errors or inaccuracies. The solution here is based on a spatial Fourier decomposition that allows fast Poisson solvers.

Moreover, in 2D imaging, the continuity equation is not necessarily exactly satisfied because of the cross-plane motion. We use the approach of solving Equation (A5) and apply the correction Equation (A3) to the transversal component only without correcting the radial velocity from the Doppler measurement. This solution is equivalent to assuming the presence of a cross-plane inflow/outflow that exactly replaces the neglected radial contribution.

The solution using this approach is the result of an elliptic equation; this means that all points of space are connected and that the solution presents a mathematical regularity. The solution is continuously differentiable in all directions at all points in space.

Inclusion ad exclusion criteria

The healthy subjects were volunteers who consented to undergo echocardiography solely for this study. They were defined as asymptomatic individuals, aged ≥20 years, with no cardiovascular risk factors including genetic/family history for a cardiac disease, without a history of any cardiovascular disease or any systemic diseases known to affect the cardiovascular system, not currently on

medical therapy with cardio-active drugs, as also with normal physical cardiac examination, a normal standard surface electrocardiogram and a normal echocardiography.

Exclusion criteria comprised non-conformity to study protocol, and in particular: age ≥80 years, arterial hypertension [blood pressure >140/90 mmHg], heart rate >100 bpm or <40 bpm, diabetes mellitus [fasting blood sugar >100 mg/dl and/or HbA1c >6.5%], obesity, dyslipidemia (total cholesterol >190 mg/dl), history of coronary artery disease, previous acute myocardial infarction, stroke and transient ischemic events, valvular heart diseases, congestive heart failure, primary cardiomyopathies, congenital heart diseases, systemic diseases, cardiac pharmacologic therapies, atrial fibrillation or history of heart rhythm disorders.

Standard echocardiographic examinations and measurements

Images were acquired with subjects in the left lateral decubitus position at hold end-expiration. Left ventricular (LV) end-diastolic diameter, interventricular septum and posterior wall thickness were measured on the parasternal long-axis view using the leading edge-to-leading edge approach. LV mass was calculated using the American Society of Echocardiography formula. The LV cavity length was measured in the apical 4-chamber view at end-diastole. LV volumes and ejection fraction were calculated from the apical 4- and 2-chamber views using the biplane Simpson's method. Stroke volume was calculated as the difference between LV end-diastolic and systolic volume, divided by the body surface area. Sphericity index was calculated as the ratio between the LV internal end-diastolic diameter and the end-diastolic cavity length. Assessment of LV diastolic function was based on the transmitral peak E- and A-wave inflow velocities recorded by pulsed wave Doppler and the average peak e' wave recorded at the mitral annulus by pulsed wave tissue Doppler. Peak E-wave/peak A-wave (E/A) ratio and average peak E wave/peak e' wave (E/e') ratio were calculated. Aortic and mitral time-velocity integral ware measured on the respective Doppler traces obtained

using pulsed wave Doppler. Left atrial maximal volume was obtained using the biplane Simpson's method. Right ventricular end-diastolic and end-systolic areas were measured in the apical 4-chamber view and fractional area change was calculated. Tricuspid annular plane systolic excursion (TAPSE) was measured using M-mode echocardiography in the apical 4-chamber view to determine the right ventricular longitudinal function. Right atrial maximal volume was obtained using the monoplane Simpson's method. Each Doppler-based and M-mode measurements were obtained as the average of measurements performed in three cardiac cycles. The Mosteller formula for body surface area was used for indexation.

Center	Subjects enrolled by the Centers	Subjects considered for study	Subjects excluded from study	Reasons for exclusion		
				Anthropometric and functional data non conform to study protocol	Standard echocardiography non conform to study protocol	Both anthropometric and echocardiographic data non conform to study protocol
1	40	39	1	-	1	-
2	42	42	-	-	-	-
3	33	32	1	-	1	-
4	35	30	5	1	4	-
5	33	22	11	-	11	-
6	21	18	3	1	2	-
7	21	20	1	-	1	-
8	27	27	-	-	-	-
9	47	36	11	2	7	2
10	40	37	3	-	3	-
11	46	46	-	-	-	-
12	37	30	7	2	5	-
13	45	44	1	1	-	-
**Total**	**467**	**423**	**44**	**7**	**35**	**2**

Supplementary table 1. Subjects excluded for non-conformity of anthropometric, functional and standard echocardiography data with study protocol.

Center	Subjects considered for study	Subjects with assessable color Doppler cineloops	Subjects with unassessable color Doppler cineloops	Reasons for not assessing color Doppler cineloops			
				Incorrect image cineloop format	Image cineloops could not be opened	Unrespected criteria for image cineloop transmission	Image cineloop content non conform to study protocol
1	39	39	-	-	-	-	-
2	42	38	4	-	-	3	1
3	32	28	4	-	1	-	3
4	30	28	2	-	2	-	-
5	22	0	22	1	-	21	-
6	18	10	8	-	-	-	8
7	20	19	1	-	-	-	1
8	27	26	1	-	1	-	-
9	36	27	9	-	-	8	1
10	37	0	37	-	35	-	2
11	46	46	-	-	-	-	-
12	30	15	15	-	9	2	4
13	44	41	3	-	3	-	-
**Total**	**423**	**317**	**106**	**1**	**51**	**34**	**20**

Supplementary table 2. Reasons for the missing HyperDoppler evaluation of color Doppler cineloops at the corelab in the study subjects. *Incorrect image cineloop format*: this refers to cineloops sent in non-DICOM format. *Image cineloops could not be opened*: this refers to cineloops without the companion .vtx file needed for opening or to corrupted files that did not open when double-clicked. *Unrespected criteria for image cineloop transmission*: this refers to cineloops sent without inclusion in a zipped folder, which precluded downloading. *Image cineloop content non conform to study protocol*: lack of or non-standard apical long-axis view; color Doppler sector incorrectly positioned or sized.

ECHOCARDIOGRAPHIC CHARACTERISTICS OF ALL STUDY SUBJECTS										
	OVERALL SUBJECTS			MALES			FEMALES			
	**N**	**Median**	**25th-75th Percentile**	**N**	**Median**	**25th-75th Percentile**	**N**	**Median**	**25th-75th Percentile**	**P value**
LV EDD (mm)	423	45.0	42.0 – 48.0	223	47.3	45.0 – 50.0	200	43.0	41.0 – 46.0	<0.001
LV EDD index (mm/m^2)	423	25.2	23.4 – 27.1	223	24.5	23.0 – 26.0	200	26.3	24.1 – 28.0	<0.001
IVS thickness (mm)	423	8.5	7.9 – 9.2	223	9.0	8.0 – 10.0	200	8.0	7.0 – 9.0	<0.001
PW thickness (mm)	423	8.0	7.0 – 8.8	223	8.0	8.0 – 9.0	200	7.3	7.0 – 8.0	<0.001
LV mass (g)	423	148.9	123.3 – 179.3	223	167.9	147.9 – 199.0	200	126.4	108.2 – 148.2	<0.001
LV mass index (g/m^2)	423	83.6	69.7 – 96.5	223	89.1	77.1 – 102.0	200	77.6	65.5 – 87.9	<0.001
LV ED length (mm)	413	76.3	72.0 – 82.8	217	79.9	74.4 – 84.8	196	74.8	70.0 – 79.0	<0.001
Sphericity index	413	0.6	0.5-0.6	217	0.6	0.5-0.7	196	0.6	0.5-0.6	0.011
LV EDV (ml)	423	92.0	76.0 – 110.0	223	106.8	92.0 – 120.0	200	79.5	69.8 – 90.0	<0.001
LV EDV index (ml/m^2)	423	52.2	44.2 – 59.1	223	55.4	48.6 – 62.5	200	48.8	41.9 – 54.5	<0.001
LV ESV (ml)	423	33.0	26.0 – 40.2	223	39.0	32.0 – 46.0	200	28.6	23.0 – 33.0	<0.001
LV ESV index (ml/m^2)	423	19.0	14.9 – 22.0	223	20.5	16.9 – 23.9	200	17.1	13.8 – 19.9	<0.001
SV index (ml/m^2)	423	32.8	27.9 – 38.0	223	34.7	29.3 – 40.0	200	31.1	27.1 – 35.4	<0.001
LV ejection fraction (%)	423	63.0	60.0 – 67.0	223	63.0	60.0 – 66.0	200	64.0	61.0 – 68.0	0.004
Aortic TVI (cm)	416	23.0	21.0 – 26.0	218	23.0	20.6 – 26.0	198	24.0	21.3 – 26.3	0.028
Aortic peak velocity (cm/s)	417	120.0	108.0 – 133.0	219	119.0	106.3 – 134.8	198	120.0	110.0 – 132.0	0.366
Mitral TVI (cm)	413	21.0	18.0 – 24.3	217	21.0	17.0 – 24.0	196	21.0	18.3 – 24.9	0.280
Peak E wave (cm/s)	420	77.0	65.4 – 88.0	221	76.3	63.7 – 86.1	199	78.0	66.3 – 90.0	0.073
Peak A wave (cm/s)	420	55.7	47.0 – 67.0	221	53.0	44.5 – 64.1	199	59.0	50.0 – 70.0	<0.001
E/A ratio	420	1.4	1.1 – 1.7	221	1.5	1.1 – 1.7	199	1.3	1.1 – 1.6	0.038
Peak e' wave (cm/s)	418	13.0	11.0 – 15.0	219	13.0	11.0 – 15.0	199	13.0	10.5 – 15.1	0.878
E/e' ratio	416	5.9	5.0 – 7.0	218	5.8	4.9 – 6.9	198	6.1	5.2 – 7.1	0.020
LA volume (ml)	422	38.0	30.2 – 46.8	222	40.0	33.5 – 51.1	200	35.0	28.0 – 41.0	<0.001
LA volume index (ml/m^2)	422	21.1	17.0 – 26.1	222	21.2	17.0 – 26.9	200	20.5	17.1 – 25.3	0.357
RV EDA (cm^2)	409	17.0	14.0 – 19.0	217	18.3	15.9 – 21.0	192	15.2	13.0 – 17.3	<0.001
RV EDA index (cm^2/m^2)	409	9.3	8.0 – 10.6	217	9.4	8.1 – 10.7	192	9.0	8.0 – 9.4	0.114
RV ESA (cm^2)	409	8.2	7.0 – 10.0	217	9.7	7.8 – 11.0	192	8.0	6.3 – 9.0	<0.001
RV ESA index (cm^2/m^2)	409	4.7	3.9 – 5.6	217	4.9	4.0 – 5.8	192	4.5	3.8 – 5.3	0.015
RV FAC (%)	409	49.0	44.0 – 53.0	217	48.0	44.0 – 52.0	192	50.0	45.0 – 54.0	0.036
TAPSE (mm)	421	24.0	22.2 – 27.0	221	24.5	23.0 – 27.4	200	24.0	22.0 – 26.0	0.061
RA volume (ml)	418	29.6	23.2 – 37.0	218	33.0	26.9 – 42.0	200	26.0	22.0 – 32.0	<0.001
RA volume index (ml/m^2)	418	16.6	13.3 – 20.5	218	17.2	13.6 – 21.3	200	16.1	13.2 – 19.3	0.023

Supplementary table 3. ED: end-diastolic. EDA: end-diastolic area. EDD: end-diastolic diameter. EDV: end-diastolic volume. ESA: end-systolic area. ESV: end-systolic volume. FAC: fractional area change. IVS: inter-ventricular septum. LA: left atrium. LV: left ventricle. PW: posterior wall. RA: right atrium. RV: right ventricle. SV: stroke volume. TAPSE: tricuspid annular plane systolic excursion. TVI: time velocity integral.

	OUTLIERS OF CORELAB MEASUREMENTS					OUTLIERS OF ON-SITE MEASUREMENTS				
**Center**	**Area**	**Length**	**Depth**	**Intensity**	**gKED**	**Area**	**Length**	**Depth**	**Intensity**	**gKED**
1	-	2	-	-	-	-	-	-	-	-
2	2	1	-	-	-	-	-	-	-	-
3	-	1	-	2	-	3	3	5	2	-
4	-	1	1	-	-	-	4	5	-	-
5	-	-	-	-	-	-	2	2	-	-
6	-	-	-	-	-	-	-	-	-	1
7	-	1	-	-	2	-	-	-	-	-
8	-	1	1	-	1	2	-	-	-	2
9	-	1	-	-	-	-	2	-	-	1
10	-	-	-	-	-	-	1	1	-	3
11	-	-	-	-	-	-	-	-	-	-
12	-	-	-	-	5	-	-	-	-	8
13	-	-	-	-	-	-	1	1	1	1
**Total**	**2**	**8**	**2**	**2**	**8**	**5**	**13**	**14**	**3**	**16**
**Percentage (%)**	0.63	2.52	0.63	0.63	2.52	1.19	3.10	3.34	0.72	3.82
**P value**	-	-	-	-	-	0.437	0.641	0.013	0.889	0.328

Supplementary table 4. Number and percentage of the outlier values of the HyperDoppler measurements performed by the corelab and on-site. gKED: global kinetic energy dissipation. P values: on-site vs. corelab.

HYPERDOPPLER MEASURES				
	**N**	**Mean**	**Standard deviation**	**Coefficient of variation**
**CORELAB**				
Vortex area	315	24.4 (%)	4.8 (%)	19.7 (%)
Vortex length	309	57.4 (%)	7.4 (%)	12.9 (%)
Vortex depth	315	34.3 (%)	5.6 (%)	16.3 (%)
Vortex intensity	315	-31.5 (%)	-5.8 (%)	18.4 (%)
gKED	309	0.58	0.19	32.7 (%)
**ON-SITE**				
Vortex area	414	24.0 (%)	5.5 (%)	22.9 (%)
Vortex length	406	57.1 (%)	9.9 (%)	17.3 (%)
Vortex depth	405	33.4 (%)	6.8 (%)	20.4 (%)
Vortex intensity	416	-28.2 (%)	-6.3 (%)	22.3 (%)
gKED	403	0.65	0.24	36.9 (%)

Supplementary table 5. gKED: global kinetic energy dissipation.

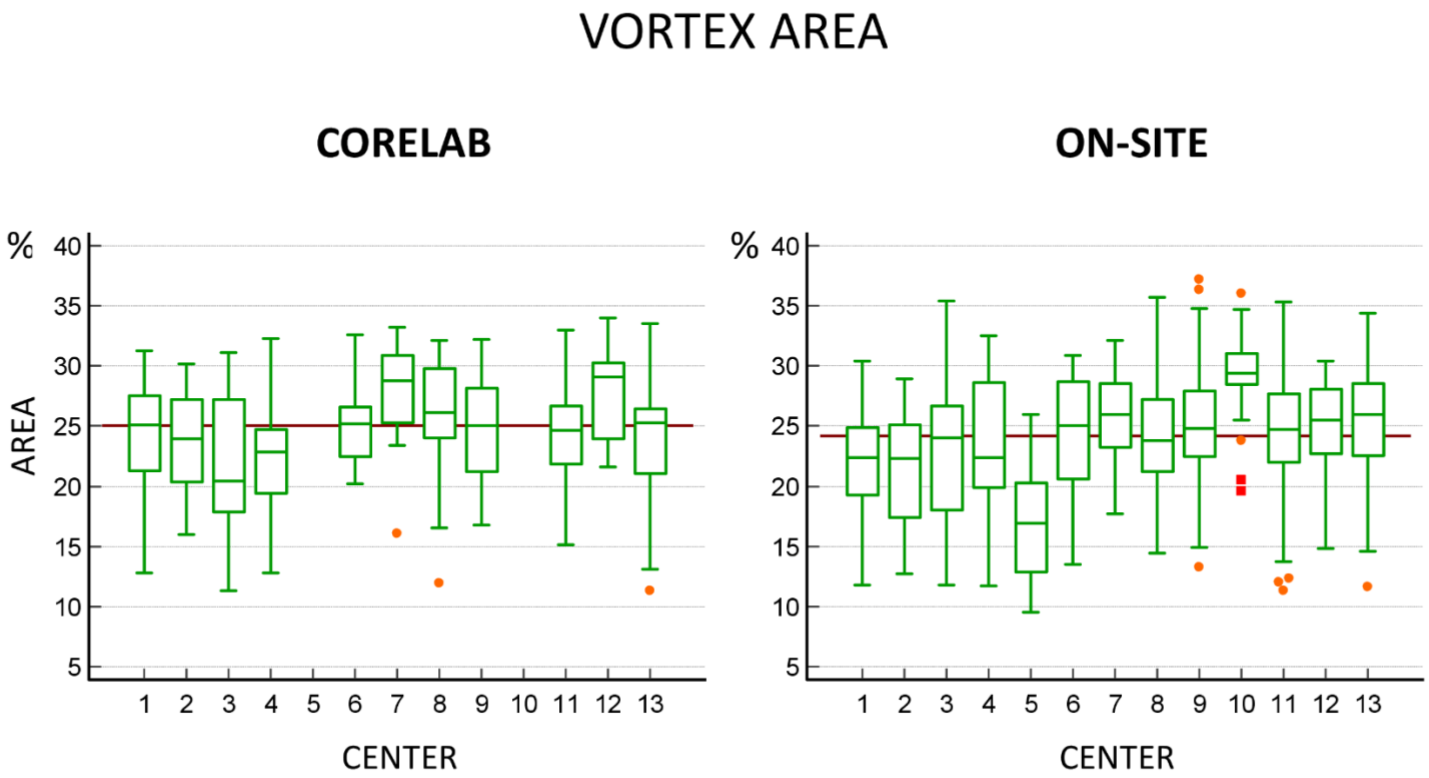

Supplementary Figure 1. Box-and-whisker plots of vortex area measured in individual Centers. For each plot, the median value, upper and lower quartiles, and upper and lower extreme values are shown. Outside values are represented with small red dots and far out values with small red squares. The red horizontal line represents the average median value.

VORTEX AREA						
Center	Corelab measurements			On-site measurements		
	N	Median (%)	25th – 75th Percentile (%)	N	Median (%)	25th – 75th Percentile (%)
1	39	25.1	21.3 – 27.5	37	22.4	19.3 – 24.9
2	36	24.0	20.4 – 27.3	42	22.3	17.4 – 25.1
3	28	20.4	17.9 – 27.3	29	24.0	18.0 – 26.7
4	28	22.9	19.5 – 24.7	30	22.4	19.9 – 28.6
5	-	-	-	22	17.0	12.9 – 20.3
6	10	25.2	22.5 – 26.6	18	25.1	20.6 – 28.7
7	19	28.8	25.3 – 30.9	19	26.0	23.3 – 28.5
8	26	26.1	24.0 – 29.8	25	23.8	21.2 – 27.2
9	27	25.0	21.2 – 28.1	36	24.8	22.5 – 28.0
10	-	-	-	37	29.4	28.5 – 31.0
11	46	24.7	21.8 – 26.7	46	24.7	22.0 – 27.7
12	15	29.1	23.9 – 30.2	29	25.5	22.7 – 28.1
13	41	25.3	21.0 – 26.5	44	26.0	22.6 – 28.6
	P<0.001			P<0.001		

Supplementary table 6. See supplementary figure 1 for graphical representation of data.

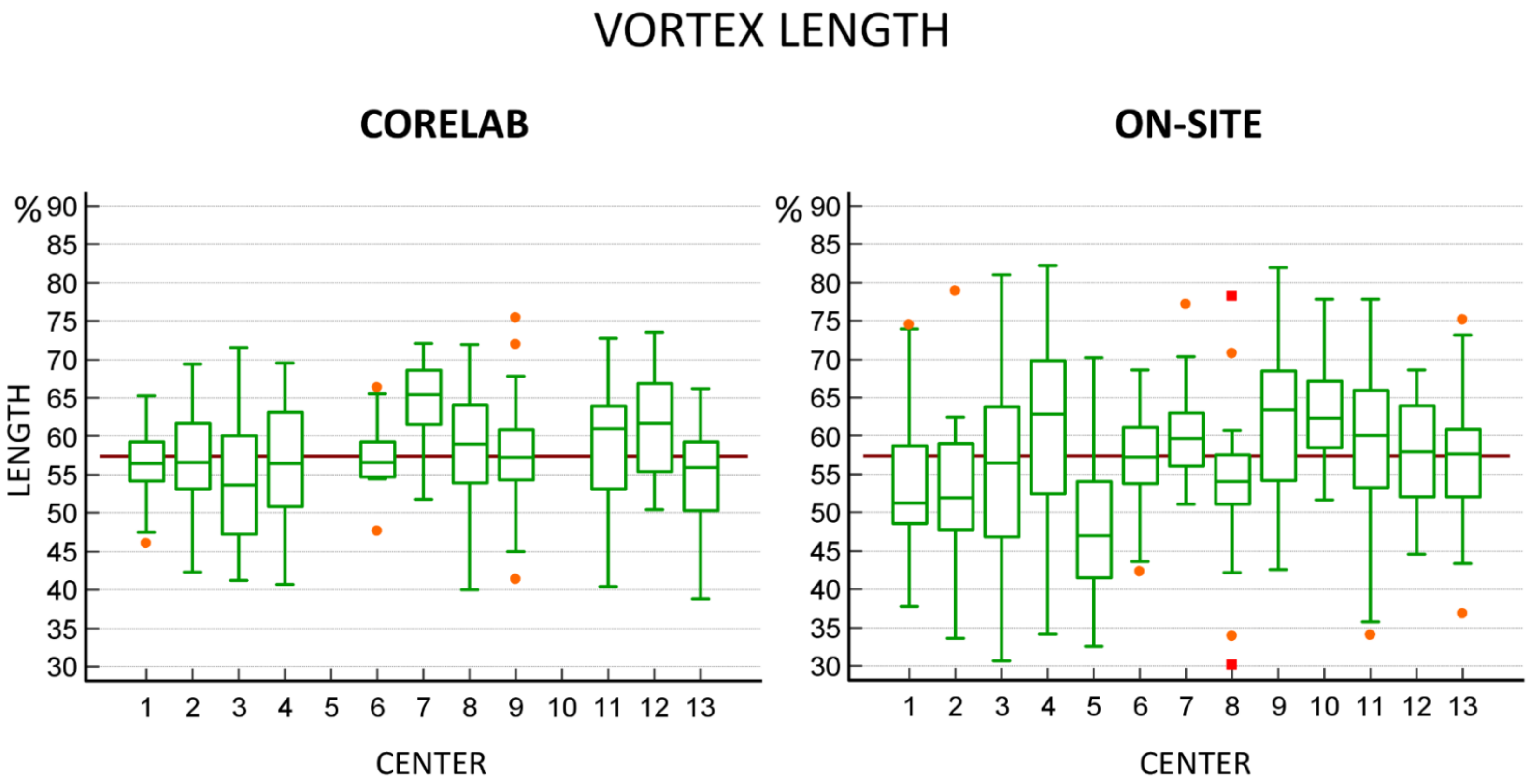

Supplementary Figure 2. Box-and-whisker plots of vortex length measured in individual Centers. For each plot, the median value, upper and lower quartiles, and upper and lower extreme values are shown. Outside values are represented with small red dots and far out values with small red squares. The red horizontal line represents the average median value.

VORTEX LENGTH						
Center	Corelab measurements			On-site measurements		
	N	Median (%)	25th – 75th Percentile (%)	N	Median (%)	25th – 75th Percentile (%)
1	37	56.5	54.1 – 59.2	37	51.2	48.6 – 58.7
2	37	56.6	53.2 – 61.7	42	51.9	47.8 – 59.0
3	27	53.6	47.2 – 60.0	29	56.4	46.9 – 63.9
4	27	56.5	50.9 – 63.2	26	62.9	52.4 – 69.8
5	-	-	-	20	47.0	41.5 – 54.1
6	10	56.6	54.7 – 59.2	18	57.3	53.8 – 61.1
7	18	65.4	61.5 – 68.6	19	59.7	56.1 – 63.0
8	25	59.0	53.9 – 64.1	27	54.1	51.2 – 57.5
9	26	57.3	54.3 – 60.9	34	63.5	54.2 – 68.5
10	-	-	-	36	62.4	58.5 – 67.1
11	46	61.0	53.1 – 63.9	46	60.1	53.3 – 66.0
12	15	61.7	55.4 – 66.8	29	57.9	52.0 – 64.0
13	41	55.9	50.4 – 59.2	43	57.7	52.1 – 60.9
	P=0.001			P<0.001		

Supplementary table 7. See supplementary figure 2 for graphical representation of data.

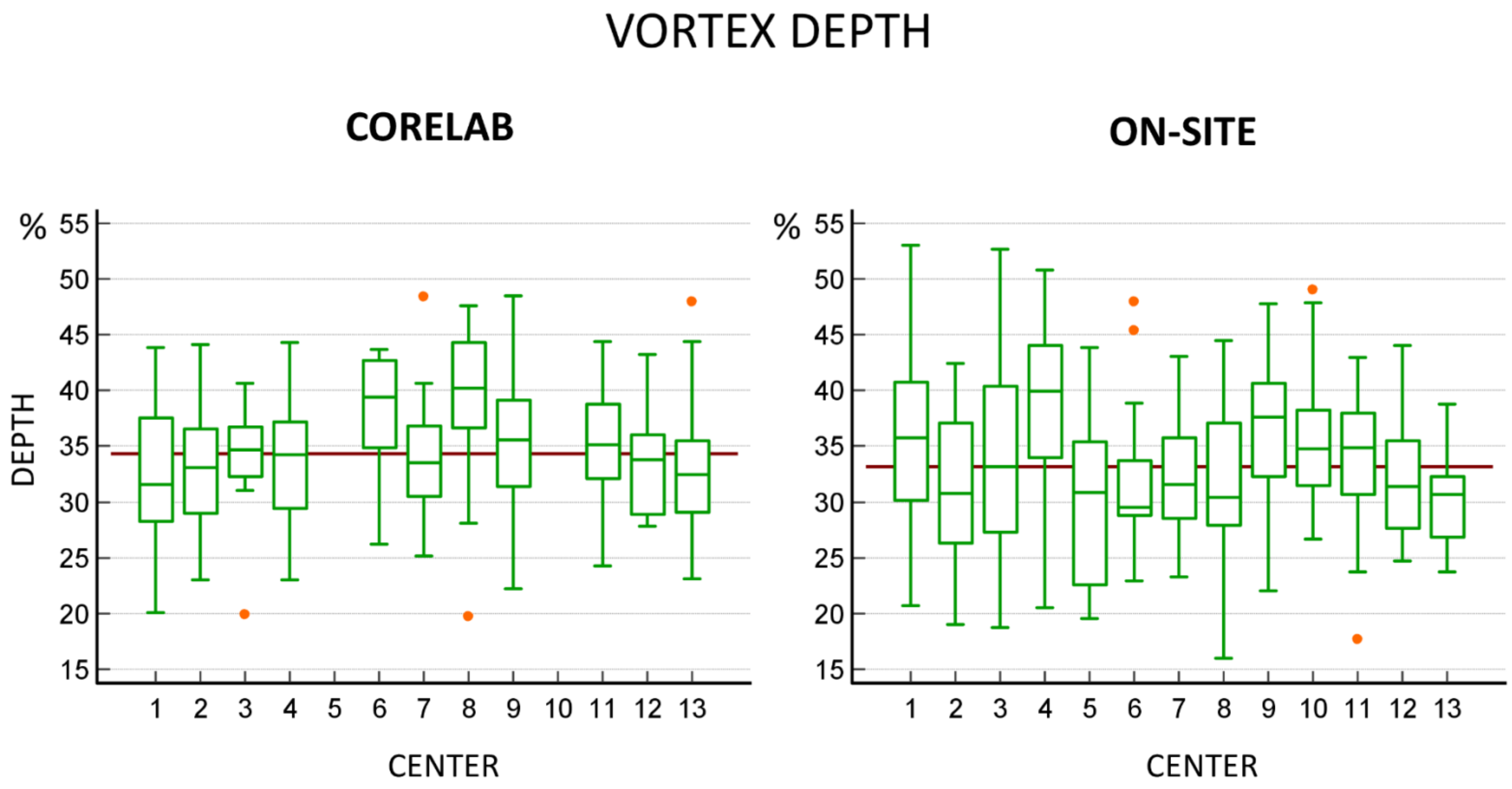

Supplementary Figure 3. Box-and-whisker plots of vortex depth measured in individual Centers. For each plot, the median value, upper and lower quartiles, and upper and lower extreme values are shown. Outside values are represented with small red dots. The red horizontal line represents the average median value.

VORTEX DEPTH						
Center	Corelab measurements			On-site measurements		
	N	Median (%)	25th – 75th Percentile (%)	N	Median (%)	25th – 75th Percentile (%)
1	39	31.6	28.3 – 37.6	37	35.8	30.1 – 40.7
2	38	33.1	29.0 – 36.6	42	30.8	26.3 – 37.1
3	28	34.7	32.3 – 36.7	27	33.2	27.3 – 40.4
4	27	34.2	29.4 – 37.2	25	39.9	34.0 – 44.0
5	-	-	-	20	30.9	22.9 – 35.4
6	10	39.5	34.9 – 42.7	18	29.5	28.8 – 33.7
7	19	33.5	30.5 – 36.8	19	31.6	28.6 – 35.8
8	25	40.2	36.6 – 44.3	27	30.2	27.9 – 37.1
9	27	35.6	31.4 – 39.1	36	37.6	32.3 – 40.7
10	-	-	-	36	34.8	31.5 – 38.3
11	46	35.1	32.1 – 38.8	46	34.9	30.7 – 38.0
12	15	33.8	28.9 – 36.0	29	31.4	27.6 – 35.5
13	41	32.5	29.1 – 35.5	43	30.7	26.8 – 32.3
		P<0.001			P<0.001	

Supplementary table 8. See supplementary figure 3 for graphical representation of data.

VORTEX INTENSITY

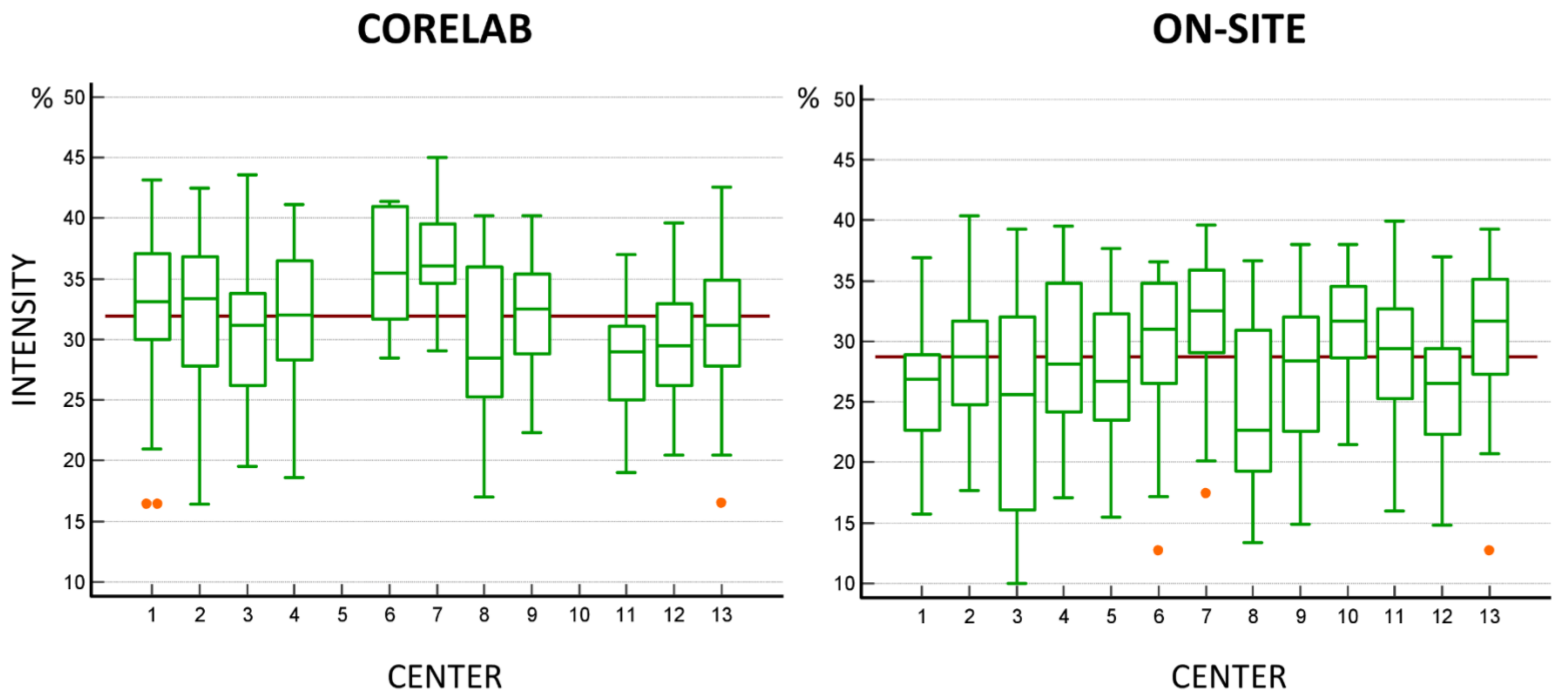

Supplementary Figure 4. Box-and-whisker plots of vortex intensity measured in individual Centers. For each plot, the median value, upper and lower quartiles, and upper and lower extreme values are shown. Outside values are represented with small red dots. The red horizontal line represents the average median value.

VORTEX INTENSITY						
Center	Corelab measurements			On-site measurements		
	N	Median (%)	25th – 75th Percentile (%)	N	Median (%)	25th – 75th Percentile (%)
1	39	-33.1	-30.0 – -37.1	37	-26.9	-22.6 – -28.9
2	38	-33.4	-27.8 – -36.8	42	-28.8	-24.8 – -31.7
3	26	-31.2	-26.2 – -33.8	30	-25.6	-16.1 – -32.0
4	28	-32.0	-28.4 – -36.5	30	-28.2	-24.2 – -34.8
5	-	-	-	22	-26.8	-23.5 – -32.3
6	10	-35.5	-31.7 – -41.0	18	-31.0	-26.5 – -34.8
7	19	-36.1	-34.6 – -39.5	19	-32.5	-29.1 – -35.9
8	26	-28.5	-25.3 – -36.0	27	-22.7	-19.3 – -30.9
9	27	-32.5	-28.9 – -35.4	36	-28.4	-22.6 – -32.0
10	-	-	-	37	-31.7	-28.6 – -34.5
11	46	-29.0	-25.0 – -31.1	46	-29.4	-25.3 – -32.7
12	15	-29.5	-26.2 – -33.0	29	-26.5	-22.3 – -29.5
13	41	-31.2	-27.8 – -34.9	43	-31.7	-27.3 – -35.2
		P<0.001			P<0.001	

Supplementary table 9. See supplementary figure 4 for graphical representation of data.

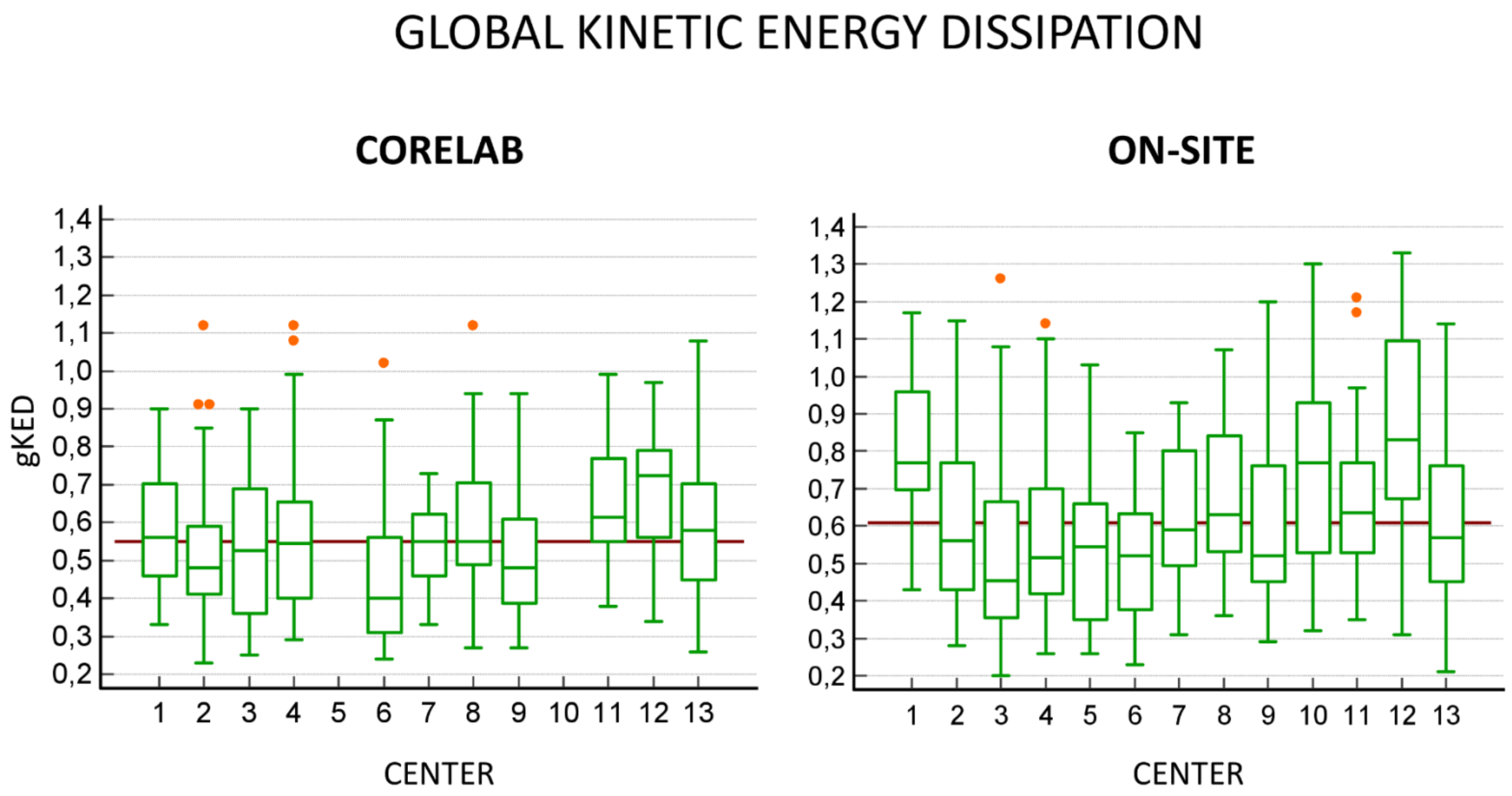

Supplementary Figure 5. Box-and-whisker plots of global kinetic energy dissipation (gKED) measured in individual Centers. For each plot, the median value, upper and lower quartiles, and upper and lower extreme values are shown. Outside values are represented with small red dots. The red horizontal line represents the average median value.

GLOBAL KINETIC ENERGY DISSIPATION						
Center	Corelab measurements			On-site measurements		
	N	Median	$25^{th} – 75^{th}$ Percentile	N	Median	$25^{th} – 75^{th}$ Percentile
1	39	0.56	0.46 – 0.70	37	0.77	0.70 – 0.96
2	38	0.48	0.41 – 0.59	42	0.56	0.43 – 0.77
3	28	0.53	0.36 – 0.69	32	0.46	0.36 – 0.67
4	28	0.55	0.40 – 0.66	30	0.52	0.42 – 0.70
5	-	-	-	22	0.55	0.35 – 0.66
6	10	0.40	0.31 – 0.56	17	0.52	0.38 – 0.63
7	17	0.55	0.46 – 0.62	19	0.59	0.50 – 0.80
8	25	0.55	0.49 – 0.71	25	0.63	0.53 – 0.84
9	27	0.48	0.39 – 0.61	35	0.52	0.45 – 0.76
10	-	-	-	34	0.77	0.53 – 0.93
11	46	0.62	0.55 – 0.77	46	0.64	0.53 – 0.77
12	10	0.73	0.56 – 0.79	21	0.83	0.67 – 1.10
13	41	0.58	0.45 – 0.70	43	0.57	0.45 – 0.76
	P=0.001			P<0.001		

Supplementary table 10. See supplementary figure 5 for graphical representation of data.

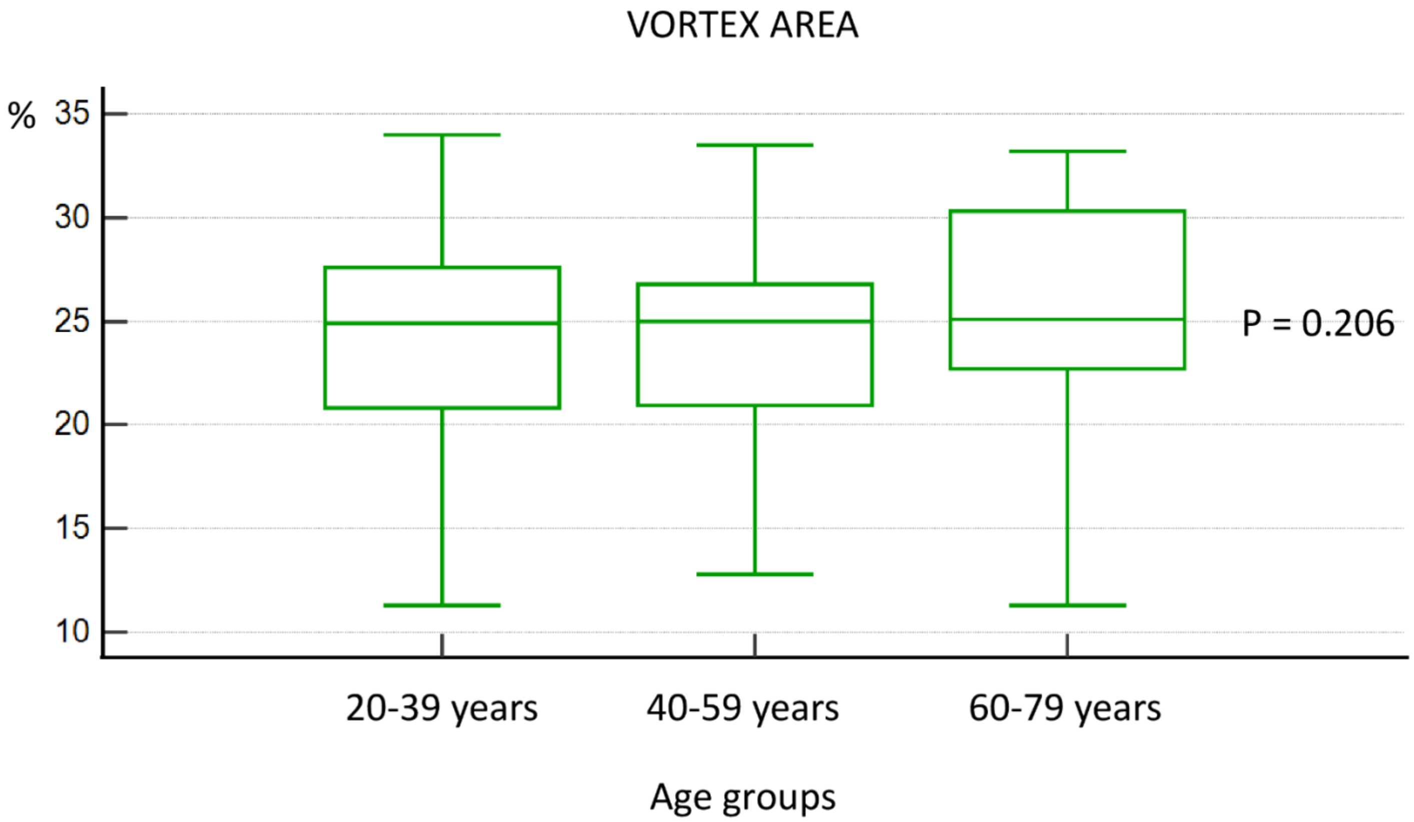

Supplementary Figure 6. Box-and-whisker plots of vortex area in the three age groups. For each plot, the median value, upper and lower quartiles, and upper and lower extreme values are shown.

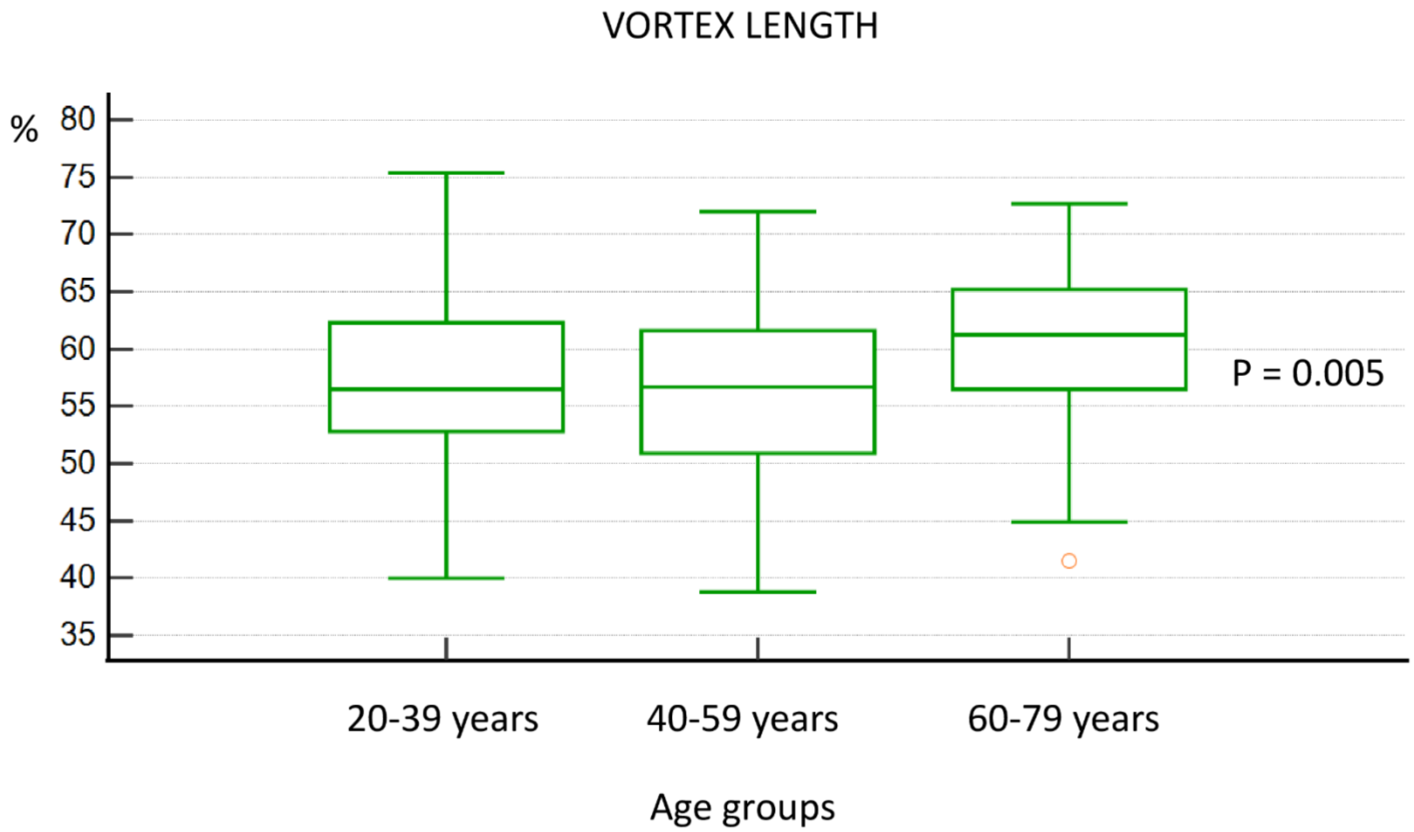

Supplementary Figure 7. Box-and-whisker plots of vortex length in the three age groups. For each plot, the median value, upper and lower quartiles, and upper and lower extreme values are shown. The outside value is represented with small red circle.

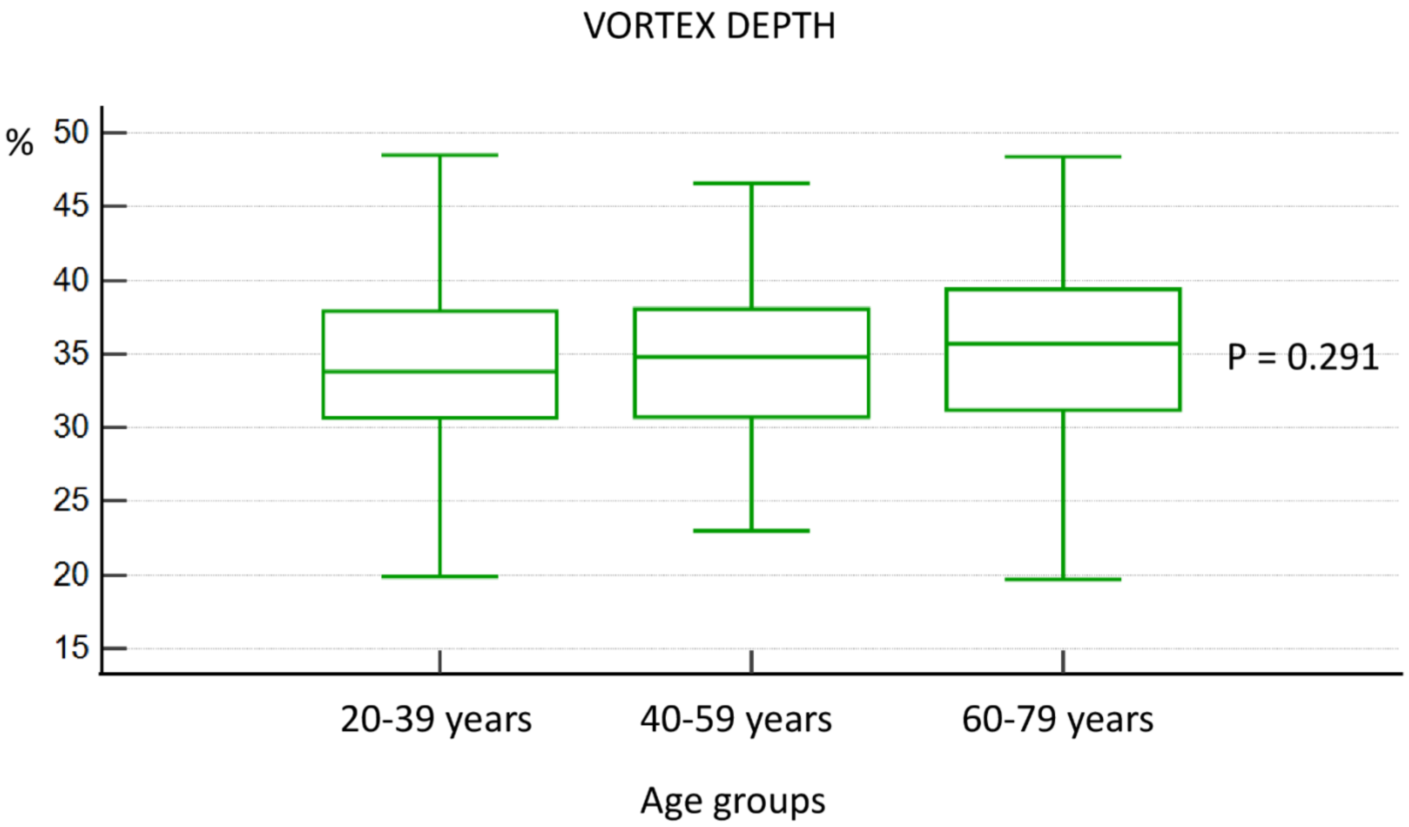

Supplementary Figure 8. Box-and-whisker plots of vortex depth in the three age groups. For each plot, the median value, upper and lower quartiles, and upper and lower extreme values are shown.

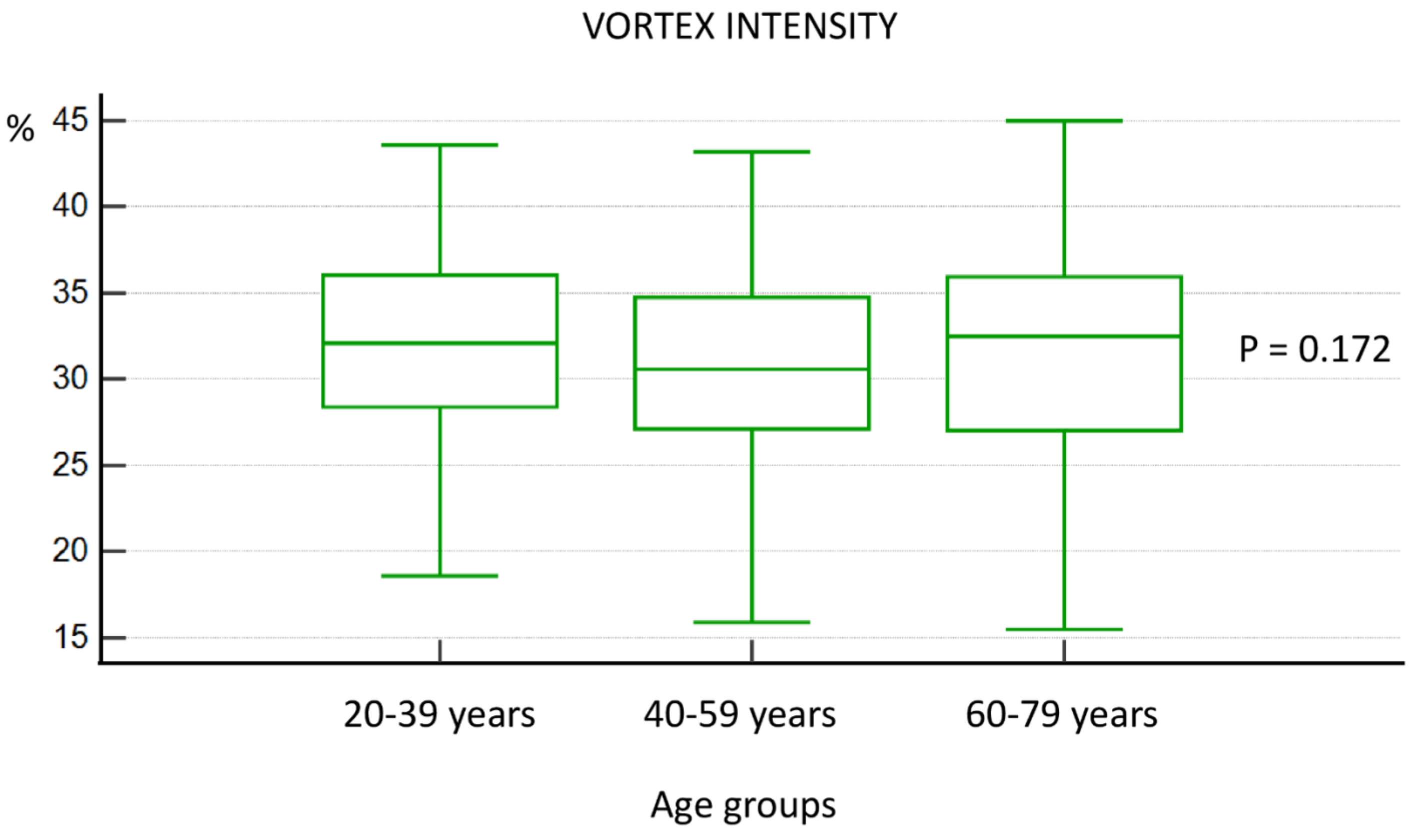

Supplementary Figure 9. Box-and-whisker plots of vortex intensity in the three age groups. For each plot, the median value, upper and lower quartiles, and upper and lower extreme values are shown.

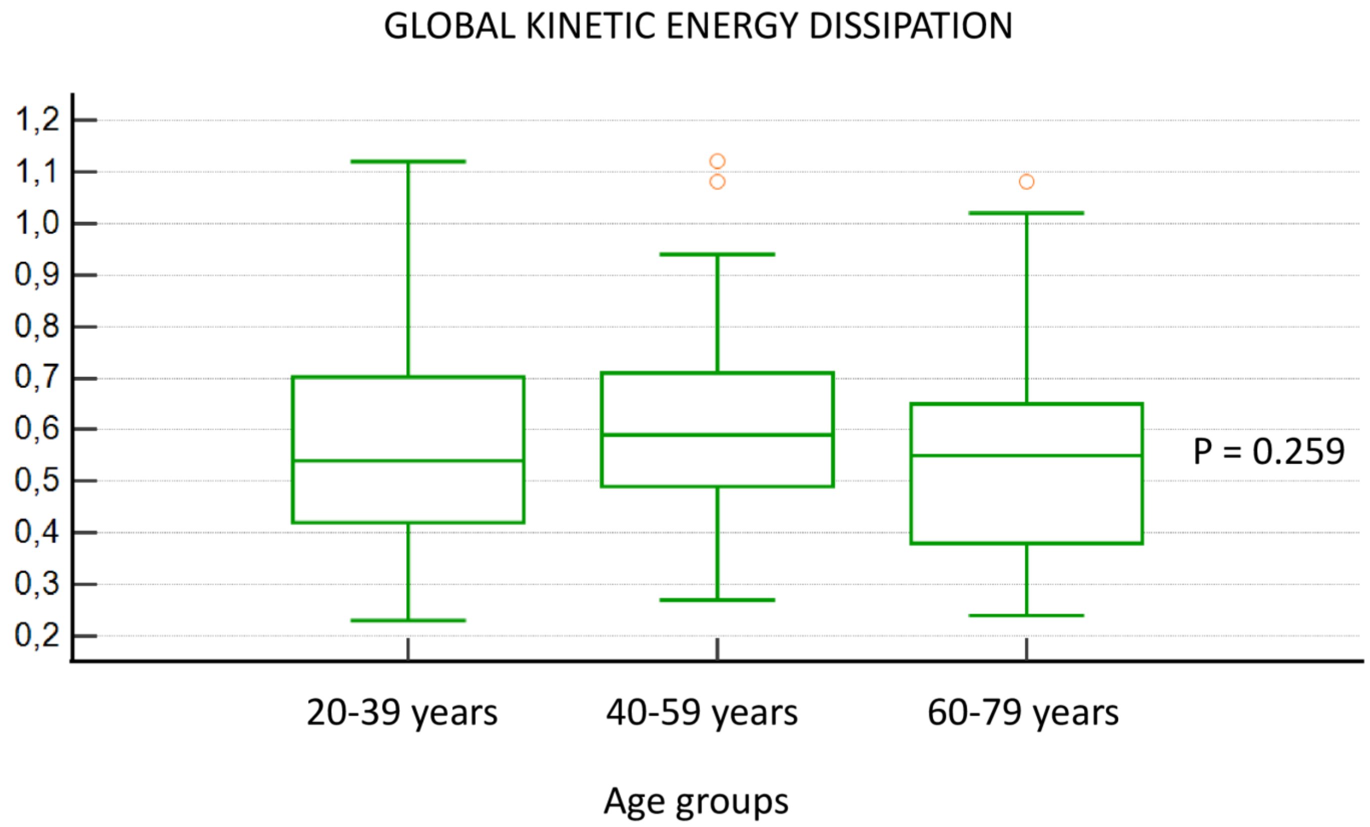

Supplementary Figure 10. Box-and-whisker plots of global kinetic energy dissipation in the three age groups. For each plot, the median value, upper and lower quartiles, and upper and lower extreme values are shown. Outside values are represented with small red circles.

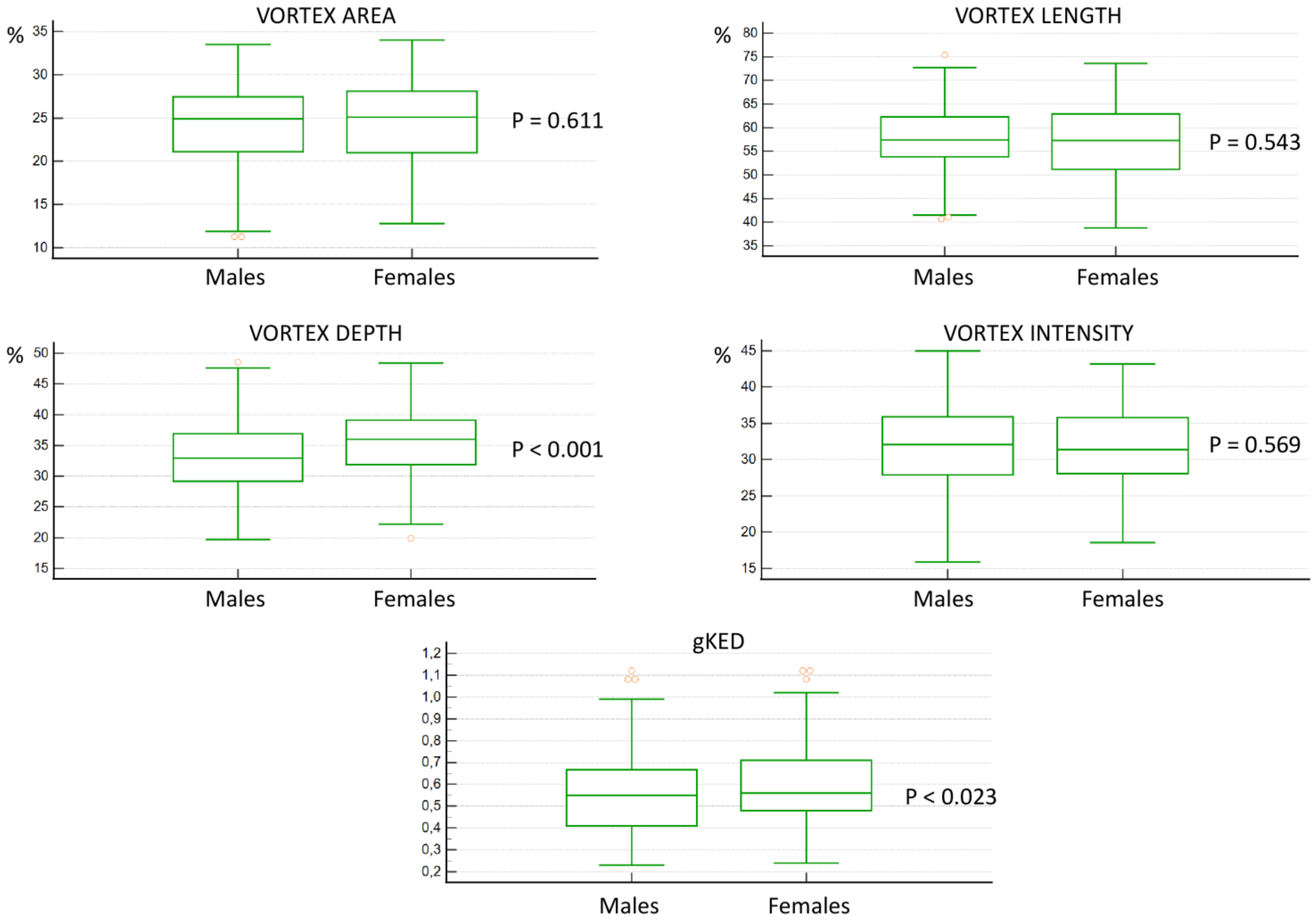

Supplementary Figure 11. Box-and-whisker plots showing values of HyperDoppler measures in males and females. For each plot, the median value, upper and lower quartiles, and upper and lower extreme values are shown. Outside values are represented with small red circles. gKED: global kinetic energy dissipation.

EVALUATION OF HYPERDOPPLER REPRODUCIBILITY					
**HyperDoppler measure**	**N of subjects**	**N of raters**	**Type of ICC**	**ICC**	**95% Confidence Interval**
Vortex Area	32	2	Consistency	0.971	0.940 – 0.986
			Absolute agreement	0.964	0.911 – 0.984
Vortex Length	32	2	Consistency	0.963	0.924 – 0.982
			Absolute agreement	0.950	0.847 – 0.979
Vortex Depth	32	2	Consistency	0.967	0.933 – 0.984
			Absolute agreement	0.964	0.924 – 0.983
Vortex Intensity	32	2	Consistency	0.972	0.943 – 0.986
			Absolute agreement	0.967	0.921 – 0.985
gKED	32	2	Consistency	0.982	0.963 – 0.991
			Absolute agreement	0.979	0.951 – 0.990

Supplementary table 11. Corelab HyperDoppler reproducibility. gKED: global kinetic energy dissipation. ICC: intraclass correlation coefficient.

REPRODUCIBILITY - VORTEX AREA

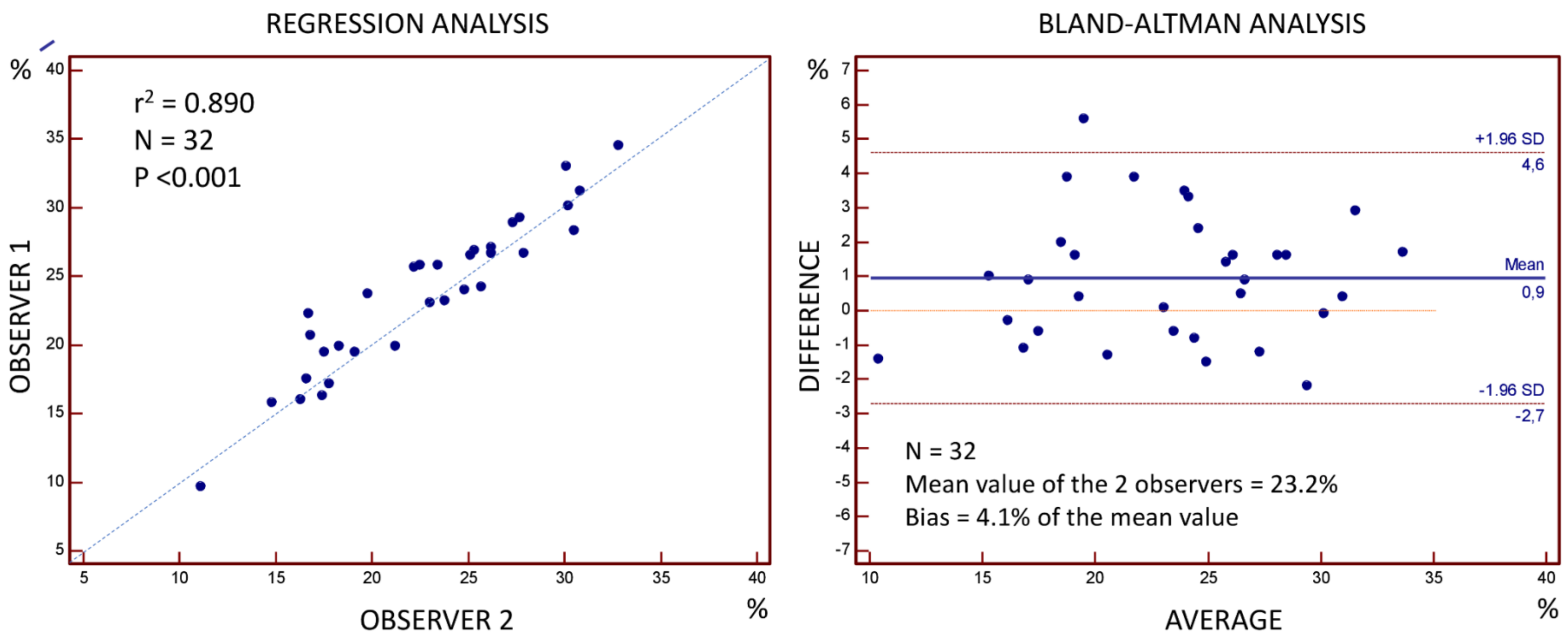

Supplementary Figure 12. Corelab reproducibility for HyperDoppler vortex area. *Left panel*. Regression analysis. The line of identity is shown. *Right panel*. Bland-Altman analysis. SD: standard deviation.

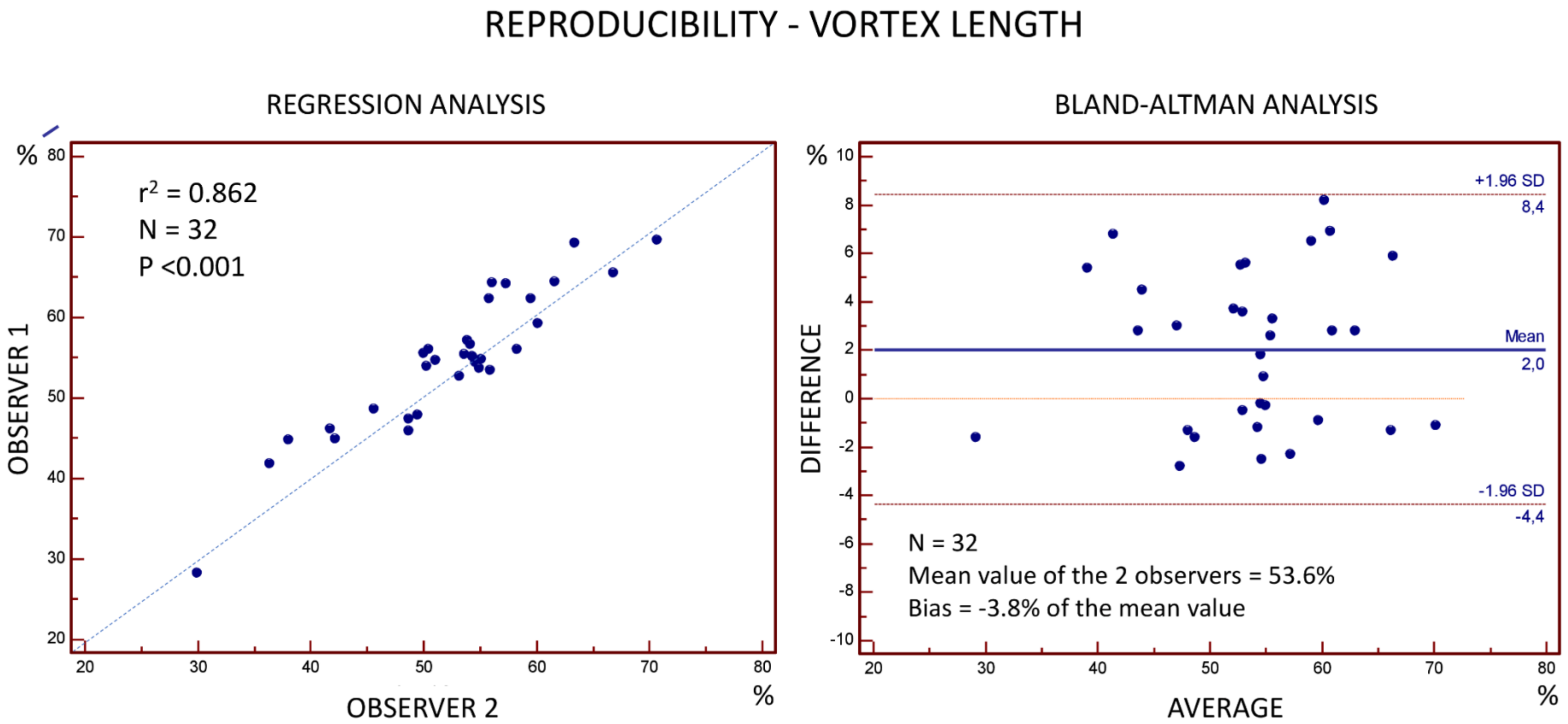

Supplementary Figure 13. Corelab reproducibility for HyperDoppler vortex length. *Left panel*. Regression analysis. The line of identity is shown. *Right panel*. Bland-Altman analysis. SD: standard deviation.

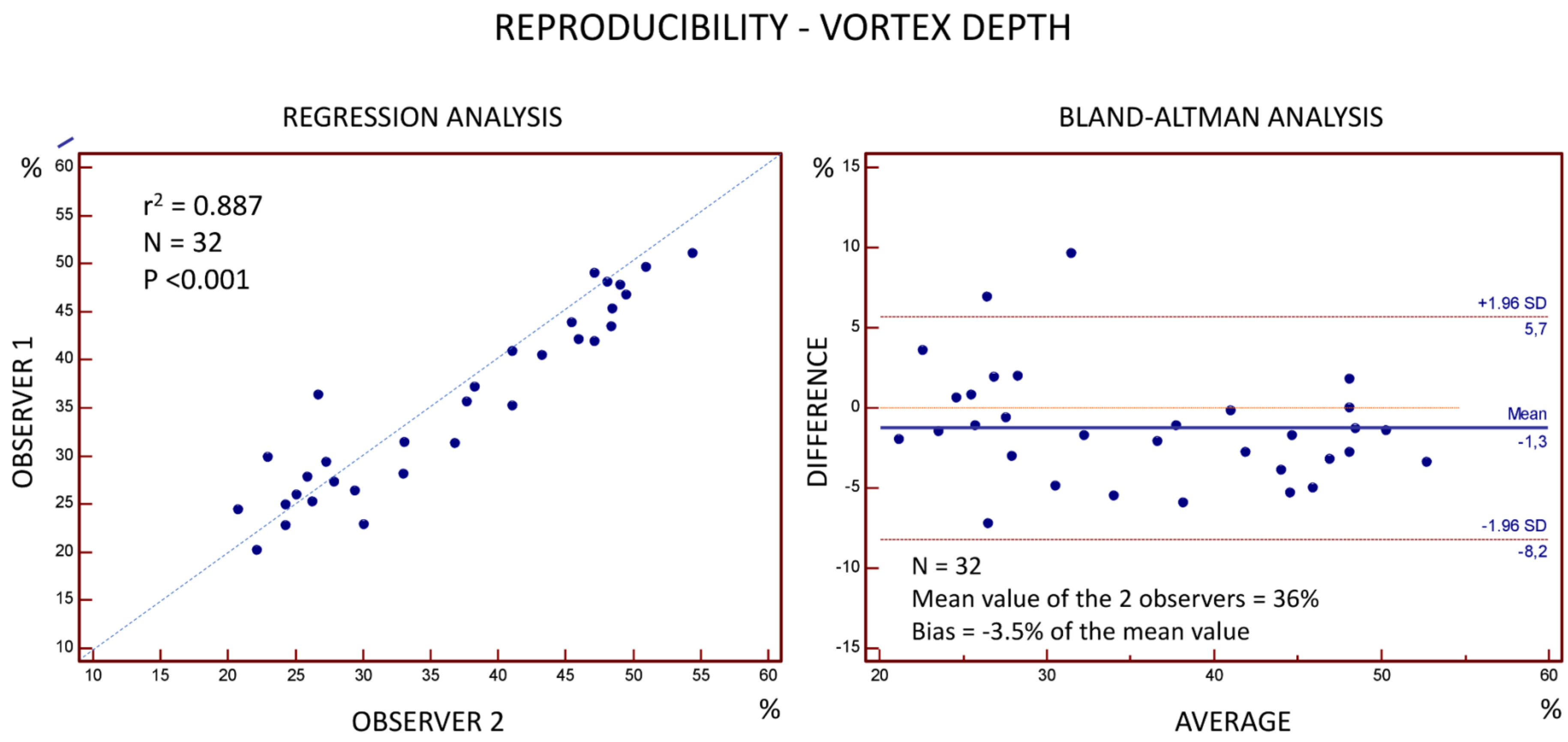

Supplementary Figure 14. Corelab reproducibility for HyperDoppler vortex depth. *Left panel*. Regression analysis. The line of identity is shown. *Right panel*. Bland-Altman analysis. SD: standard deviation.

REPRODUCIBILITY - VORTEX INTENSITY

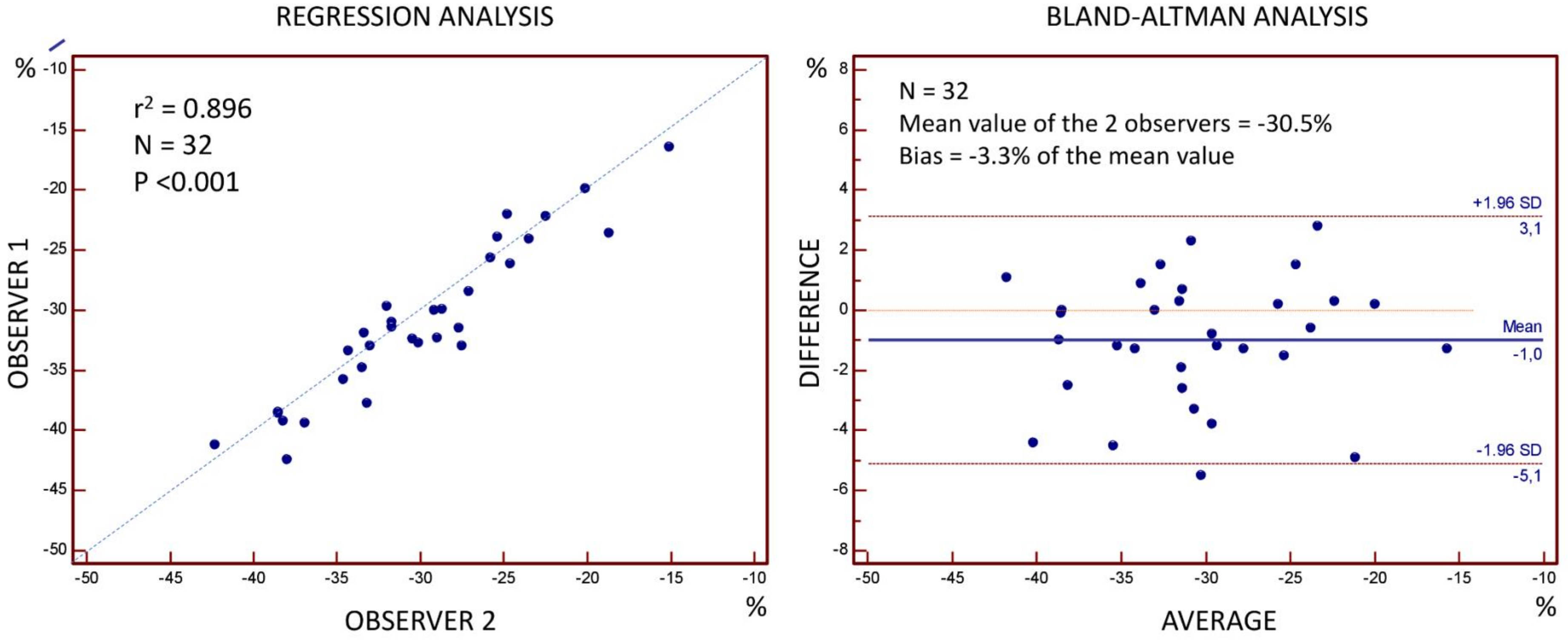

Supplementary Figure 15. Corelab reproducibility for HyperDoppler vortex intensity. *Left panel*. Regression analysis. The line of identity is shown. *Right panel*. Bland-Altman analysis. SD: standard deviation.

REPRODUCIBILITY - GLOBAL KINETIC ENERGY DISSIPATION

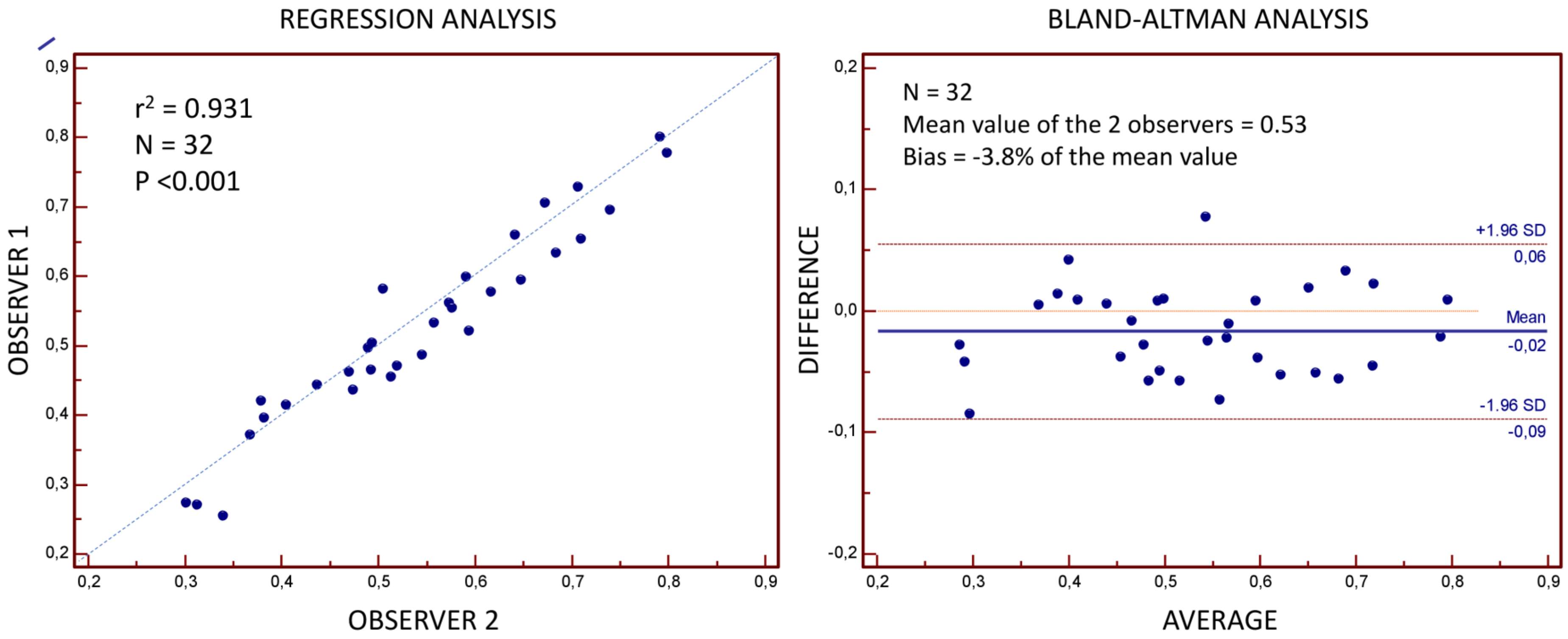

Supplementary Figure 16. Corelab reproducibility for HyperDoppler global kinetic energy dissipation. *Left panel*. Regression analysis. The line of identity is shown. *Right panel*. Bland-Altman analysis. SD: standard deviation.
